# Adducts of the Zinc Salt of Dinitramic Acid

**DOI:** 10.3390/ma16010070

**Published:** 2022-12-21

**Authors:** Sergey G. Il’yasov, Vera S. Glukhacheva, Dmitri S. Il’yasov, Egor E. Zhukov, Ilia V. Eltsov, Andrey A. Nefedov, Yuri V. Gatilov

**Affiliations:** 1 Institute for Problems of Chemical and Energetic Technologies, Siberian Branch of the Russian Academy of Sciences (IPCET SB RAS), 659322 Biysk, Russia; 2 Department of Organic Chemistry, Faculty of Natural Sciences, Novosibirsk State University, 630090 Novosibirsk, Russia; 3 Department of Physical Organic Chemistry (OPOC), Vorozhtsov Novosibirsk Institute of Organic Chemistry, Siberian Branch of the Russian Academy of Sciences (NIOCh SB RAS), 630090 Novosibirsk, Russia

**Keywords:** dinitramide, carbohydrazide, glyoxal, adducts, zinc (II), macrocycles

## Abstract

Herein, we describe the synthesis of coordination compounds starting from carbohydrazide ((H_2_NHN)_2_C=O (CHZ)) and the Zn^2+^ salt of dinitramic acid (HDN), which are high-nitrogen substances that exhibit properties similar to those of a burning-rate inhibitor of pyrotechnic compositions. This study demonstrates that these compounds react with glyoxal to furnish adducts of metal–organic macrocyclic cages bearing the elements of carbohydrazide, complexing metals and the HDN anion, depending on the ratio of the starting reactants. The assembled macrocyclic cage has “host–guest” properties and is a safe container for the storage of HDN salts. X-ray crystallographic analysis of the resultant coordination compound, [Zn(chz)_3_(N(NO_2_)_2_)_2_]), indicated that the metal–ligand association occurs via the N and O atoms of carbohydrazide. The zinc salt of dinitramic acid, which is enclosed into adducts with a macrocycle, is thermally stable and insensitive to mechanical impacts. The complex zinc salt of dinitramide was shown herein to exhibit inhibitory activity towards the burning rate of pyrotechnic compositions.

## 1. Introduction

Dinitramic acid (HDN) salts play an important role in creating high-energy materials and compositions based thereupon as regulators of the oxygen content in a formulation. The ammonium salt of dinitramic acid (and) has been recognized to be an effective oxidizer for the design of energy-rich rocket propellants. ADN is among the salts of dinitramic acid [1,2,3] that are most interesting for use as a basic green oxidizer in solid propellants [4,5,6,7] and as a stabilizing additive to ammonium nitrate [8]. Presently, studies are being conducted towards the synthesis of novel compounds based on dinitramide (DN) [9,10,11,12,13,14], which is chiefly associated with high oxygen content per unit weight and ecological safety of the product itself, as well as its combustion products and production technology. The demonstrated thermal stability of HDN-based coordination compounds as compared to the HDN anion itself has provoked an interest in investigating these salts as igniters, including those initiated by laser [15,16,17]. Compounds based on HDN and CHZ generate two types of salts in ratios of 1:1 and 2:1, respectively, whereas the nickel salt of HDN with CHZ furnishes a [Ni(chz)_3_(dn)_2_]coordination compound, where nickel is the cation, (chz) is the ligand, and (dn) is the anion (^–^N(NO_2_)_2_, a high-nitrogen substance that inhibits the burning rate of pyrotechnic formulations [18]), which is essential, in certain instances, for solid propellants to burn [19,20,21].

**Scheme 1:**
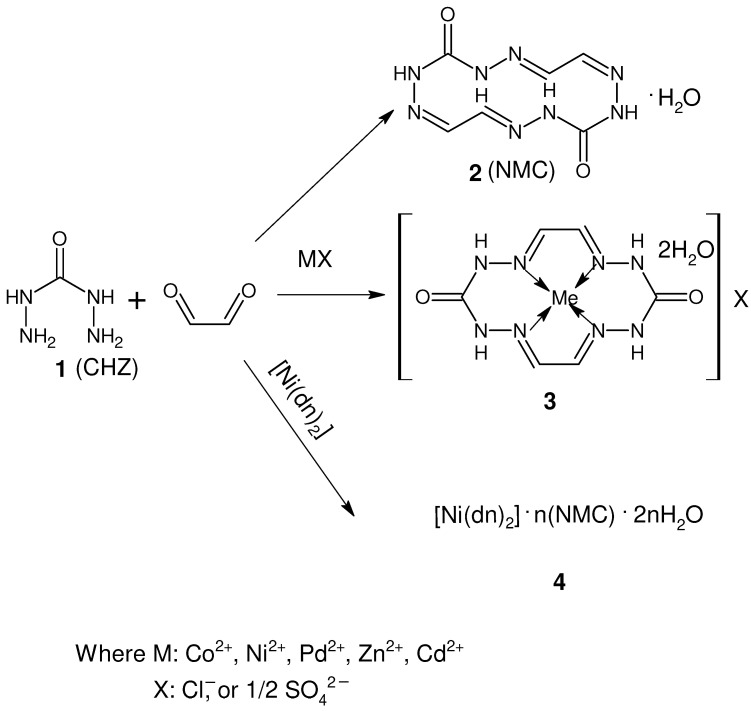
Synthesis of macrocycle **2** [26,27] and its reaction products, **3** [28] and **4** [29].

CHZ is of interest because it is often used as a ligand in the synthesis of coordination compounds and as a high-energy compound [22,23,24,25]. It easily reacts with aldehydes and ketones to form the respective hydrazones. In the reaction of CBZ with glyoxal, N-heterocyclic macrocycle (NMC) **2** (1,2,4,5,8,9,11,12-octaazacyclotetradeca-5,7,12,14-tetraene-3,7-dione) is formed [26,27], whereas in the presence of metal salts, NMC complexes of general formula **3** [28] and NMC adducts **4** [29] can be obtained (Scheme 1).

Compound **2** (NMC) can form solvates with both water and organics, such as (C_2_H_5_)_3_N, (CH_3_)_2_CHO and (C_2_H_5_)_2_O. Compound **2** is endowed with a broad spectrum of “host–guest” properties and is of interest as a building cage for the construction of diverse energetic structures, as well as in supramolecular chemistry. In this context, using a general synthesis protocol for **3**, we synthesized an adduct containing [Ni(dn)_2_] “encapsulated” into the NMC by a reaction between carbohydrazide and glyoxal in the presence of nickel dinitramide **4** (Scheme 1). This improved the physicochemical properties of the dinitramide anion by reducing its sensitivity to mechanical stimuli and enhancing its resistance to external impacts, enabling the use of this compound as a modifier of the burning rate of a pyrotechnic formulation [28]. The ability of DN to generate adducts when reacted with pyridine, morpholine, and acetonitrile was previously shown by Lukyanov et al. [3].

In this respect, we should have solved the unaddressed issues regarding the zinc cation because in our studies, we used NMR spectroscopy, which is less informative in identifying the nickel cation-based adduct. There arises a question regarding the coordination of the zinc cation with CHZ because it is impossible to obtain an unambiguous answer about the coordination number (4 or 6) if no experiment is conducted. Zn^2+^ generally exhibits a coordination number (CN) of 4, but there are examples in which the zinc cation shows a CN of 6, including those with carbohydrazide [22,23,24]. Such behavior depends on the nature of the anion. Information on the synthesis and properties of zinc dinitramide is available in Bottaro’s patent [1]. Anhydrous [Zn(dn)_2_] with a melting point (dec.) of 157–159 °C was synthesized by reacting ammonium dinitramide with diethylzinc [4]. The structural formula of the crystallohydrate [Zn(H_2_O)_6_] 2(dn)·2H_2_O prepared by reacting barium dinitramide with zinc sulfate was reported in [30]. However, the literature lacks information on the synthesis of a coordination compound starting from zinc dinitramide and carbohydrazide. In this regard, the aim of the present was to develop synthesis methods for a coordination compound based on zinc (II) dinitramide (DN) and CHZ, as well as its adducts with glyoxal.

## 2. Materials and Methods

General information. ^1^H,^13^C NMR spectra of samples were recorded on Bruker Avance III 500 and Bruker AV-400 instruments (Bruker Corporation, Billerica, MA, USA) operating at 500.03 MHz and 400.13 MHz (^1^H) and 125.73 MHz and 100.61 MHz (^13^C), respectively. Signals of DMSO-*d_6_* were employed as the internal standard: δ = 2.50 ppm for residual protons of *CH*D_2_ in ^1^H NMR and δ = 39.51 ppm for CD_3_ in ^13^C NMR, as well as signals of CDCl_3_: δ = 7.24 ppm for residual protons in ^1^H NMR spectra and δ = 77.16 ppm for ^13^C NMR. ^14^N, ^15^N NMR spectra were recorded on a Bruker Avance III 500 instrument with operating frequencies of 50.67 MHz and 36.13 MHz (^14^N and ^15^N, respectively). The chemical shifts for nitrogen nuclei were referred to formamide (external standard): δ(N) = 112.5 ppm. Two-dimensional heteronuclear ^1^H,^13^C HMBC and ^1^H,^13^C HSQC techniques were used for the structural identification and signal assignment in ^1^H,^13^C NMR.

UV spectra were obtained by a Varian Cary 50 UV/Vis instrument (Varian, Belrose, Australia) using quartz cells in water (*l* = 0.5) at 20 °C. IR spectra were recorded by an FT-801 Fourier spectrometer using KBr pellets (Simex, Novosibirsk, Russia) at 4000 to 500 cm^−1^. A CHNO FlashEATM 1112 instrument (ThermoQuest Italia S.p.A., Milan, Italy) was employed for elemental analysis. The melting temperature was measured using a Böetius PHMK (Veb Analytik, Dresden, Germany) apparatus. The temperature of decomposition was determined using TGA/SDTA 851e and DSC 822e thermal analyzers (Mettler Toledo, Zurich, Switzerland) at temperatures ranging from 25 to 300 °C and 25 to 500 °C in a nitrogen atmosphere, with a heating rate of 10 °C/min. STARe 11.0 software was used to digitize and process the results. A Thermo Electron Double Focusing System (Thermo Electron Scientific Instruments Corporation, Madison, WI, USA) was employed for mass spectrometry and molecular weight measurements. Samples in metal vials were placed into a spectrometer by direct injection; if needed, the vial with the sample can be heated within the temperature range of 25 to 360 °C. The spectrometer was operated in an electron ionization regime at 70 eV. The precise ion masses were measured with respect to standard perfluorokerosene.

Crystallography. All the measurements were performed at the Collective Chemical Service Center SB RAS (Novosibirsk, Russia). X-ray analysis of **6** was performed using a Bruker KAPPA APEX II CCD instrument (Bruker Corporation, Billerica, MA, USA) with monochromated MoKα (0.71073 Å) radiation at room temperature. Corrections for absorption were performed with SADABS [31]. The structure was identified by the direct method. Locations and temperature factors of non-hydrogen atoms were refined using the full-matrix least-squares method in an anisotropic manner. The riding model was applied to refine H atoms. All estimations were performed in SHELX-2018 programs [32]. The coordinates of atoms and temperature parameters thereof were deposited in the Cambridge Structural Database.

Crystal data for **6**: C_3_H_18_N_18_O_11_Zn**^.^**0,5(H_2_O); Mr = 556.73; monoclinic system; space group P2_1_/*c*; *a* = 12.993(3); *b* = 8.875(2); *c* = 35.138(9) Å; β = 92.739(6)°; *V* = 4047.2(17) Å^3^; *Z* = 8; *D*calc = 1.827 g·cm^−3^; μ = 1.311 mm^−1^; *θ* scan range, 1.6 < 26.0°; measured reflections, 46,823; independent, 7965 (*R*_int_ = 0.0441); observed, 6453 *I* ≥ 2σ(*I*); refined parameters, 659; *R*(*I* ≥ 2σ(*I*)) = 0.0595; *wR*_2_ = 0.1651; *S* = 1.003. CCDC 2213295 contains supplementary crystallographic data for this paper. These data can be obtained free of charge from the Cambridge Crystallographic Data Centre via www.ccdc.cam.ac.uk.

Commercial products: diaminourea **1**, a 40% aqueous glyoxal solution, potassium perchlorate (chemically pure, specification no. 6-09-3801-76; 63–160 µm size), aluminum (ASD-4 brand, specification no. 48-5-226-87; 4–10 µm size), potassium nitrate (chemically pure, Russian State Standard (GOST) No. 4217-77; 63–160 µm size), and zirconium (calciothermic Zr powder; specification no. 48-4-234-84; 1–15 µm size). 

### 2.1. Synthetic Methods

[Zn(H_2_O)_6_]·2(dn) 2H_2_O (**5**) was prepared by the method reported in [30].

#### 2.1.1. Synthesis of [Zn(chz)_3_(dn)_2_] (**6**)

Compound **5** (2.11 g, 0.005 mol) was dissolved in water (10 mL), and CHZ (1.35 g, 0.015 mol) in water (5 mL) was added with constant stirring at 20–23 °C. The mixture was then held for 30 min. The precipitate was filtered and washed with water, alcohol, and ether and dried for 24 h at 60 °C under reduced pressure. Yield: 1.46 g (53.3 %). T (dec.): 222 °C (DSC). FTIR (KBr, cm^−1^): 3321, 3266, 3166, 1644, 1605, 1536, 1430, 1344, 1204, 1178, 1088, 1031, 945, 889, 828, 762, 731, and 615. Found (%): C, 6.23; H, 3.29; N, 45.86; Zn, 12.04. Calcd (%): C, 6.57; H, 3.29; N, 46.03; Zn, 11.95 calcd. for C_3_H_18_N_18_O_11_Zn_._

#### 2.1.2. One-Pot Synthesis of Adduct **7** from **1**, **5**, and Glyoxal

Compound **5** (2.11 g, 0.005 mol) was dissolved in water (30 mL), and CHZ (0.9 g, 0.01 mol) in water (10 mL) and 40% aqueous glyoxal (0.58 g, 0.01 mol, 1.14 mL) were added with constant stirring at 20–23 °C. The mixture was then held for 60 min. The precipitate was filtered off and washed with water, alcohol, and ether. Yield: 1.6 g (19.39%). T (dec.): 101, 223, and 274 °C (DSC). FTIR (KBr, cm^−1^): 3450 (H_2_O), 3231 (NH), 2933, 1693 (C=O), 1603, 1519 (N=N→O), 1443, 1361, 1266, 1187 (N=N→O), 1120, 1020, 929, 826, 762, and 740. Found (%): C, 21.82; H, 3.81; N, 39.09; Zn, 3.99. Calcd. (%): C, 21.82; H, 4.12; N, 39.04; Zn, 3.97 calcd. for C_30_H_68_N_46_O_32_Zn_._ Calcd. FW, 1650. [Zn(dn)_2_]·5(NMC)·14H_2_O.

#### 2.1.3. Synthesis of Adduct **8** from **6** and Glyoxal

Compound **6** (2.74 g, 0.005 mol) was solubilized in water (40 mL), and a 40% glyoxal solution (1.14 mL, 0.01 mol) was then added under stirring. The reaction mixture was allowed to sand for 1 h at room temperature. The yellow-colored precipitate was filtered off and washed with water, alcohol, and ether. Yield: 1.9 g (39.1%). T (dec.): 97, 212, and 266 °C (DSC). FTIR (KBr, cm^−1^): 3500 (H_2_O), 3227 (NH), 2933, 1665 (C=O), 1616 (NH), 1523 (N=N→O), 1444, 1354, 1272, 1180 (N=N→O), 1118, 1014, 930, 826, and 756. Found (%): C, 16.49; H, 3.61; N, 40.00. Zn, 6.75. Calcd. (%): C, 16.47; H, 3.91; N, 39.39. Zn, 6.73. calcd. for C_40_H_114_N_82_O_61_Zn_3._ FW 2916. [Zn(dn)_2_]_3_·4(chz)·6(NMC)·21H_2_O.

### 2.2. Pyrotechnic Formulations

1. 70% potassium perchlorate and 30% aluminum;
2. 48% potassium nitrate and 52% zirconium.

Burning-rate modifier: compound **6**. 

### 2.3. Preparation of Ingredients

The chemicals used herein were dried to a constant weight at 100 °C. Oxidizers were additionally comminuted using a ball mill and screened through a sieve (63–160 µm).

### 2.4. Preparation of Pyrotechnic Compositions

According to the formulation, the components were weighed with 0.0001 g accuracy. An agate mortar was employed to mix the components.

### 2.5. Measurements of Burning Rate of Pyrotechnic Mixtures

The burning rate was measured on 10 mm wide cylindrical charges using ionization detectors. The passage time of the burning front was registered using an AKTAKOM ASK-3107 oscillograph (OOO Atakom, Moscow, Russia) with a 5 kHz sampling rate. 

## 3. Results and Discussion

### 3.1. Synthesis and Characterization of Compounds 

**Scheme 2:**
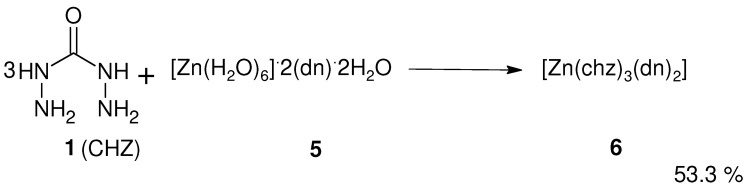
Synthesis of tris(carbohydrazide-N,O)zinc(II)dinitramide (**6**).

The tris(carbohydrazide-N,O)zinc(II)dinitramide (**6**) complex (Scheme 2) was prepared by mixing [Zn(dn)_2_] aqueous solution with carbohydrazide. 

**Figure 1:**
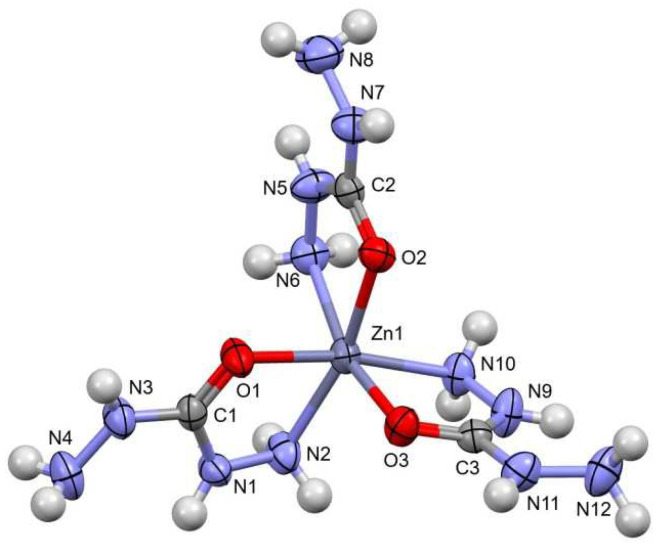
Molecular structure of **6**.

Compound **6** precipitated from the reaction mixture during the synthesis. The yield (53.3 %) of product **6** is shown in Scheme 2 only for the precipitated portion, disregarding its presence in the solution. The UV spectrum of the aqueous solution matches those of the DN salts (λ = 284 nm). The molecular structure of **6** was validated by single-crystal X-ray analysis (Figure 1). In the crystal unit cell, there are eight cations (only one is shown in Figure 1), sixteen anions (four of which are disordered), and four water molecules.

**Figure 2:**
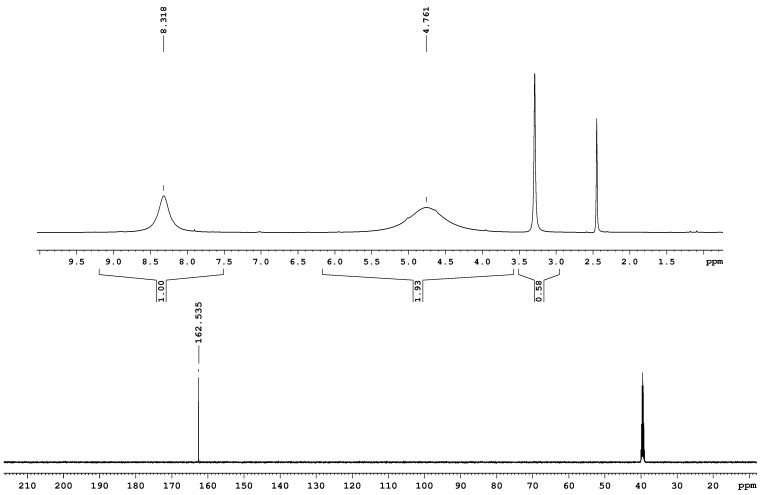
^1^H and ^13^C NMR spectra of sample **6**.

The spectrophotometric analysis (UV spectrum) of the precipitate for the DN anion showed its content to be 36.51 %, corresponding to tris(carbohydrazide-N,O)zinc(II)dinitramide dehydrate (36.3%). DTA (Supplementary Materials) showed 3.1% water removal at up to 60 °C, corresponding to the calculated water content of 1 mol for **6**. The analysis for zinc, as well as elemental analysis for C,H,N, corroborated the corresponding composition of compound **6**. This sample exhibited very good solubility in DMSO; we were able to characterize this compounds in the ^1^H, ^13^C, ^14^N, and ^15^N nuclei. The ^1^H NMR spectrum had two signals at 4.76 ppm and 8.32 ppm at an integral intensity ratio of 2:1 (Figure 2), which corresponds to the presumed structure, i.e., the presence of CHZ. 

The ^13^C NMR spectrum (Figure 2) shows only one signal (162 ppm), which also corresponds to the presumed structure. However, an attempt to acquire information on spin–spin coupling constants (SSCC) yielded no positive results; the signal was observed to broaden slightly, with no discernible separate multiplet lines.

A review of literature showed that the chemical shift of the carbon atom of the carbohydrazide carbonyl for free ligand is approx. 164.2 ppm [33]. The protonation of the one NH_2_ group causes a slight upfield shift up to 160.8 ppm [34,35,36]. Thus, it can be speculated that the observed chemical shift of 162.5 ppm may correspond to the coordinated form of carbohydrazide, as validated by X-ray diffraction (Figure 1). From this perspective, the detected signal corresponds to the sought-after structure. 

**Figure 3:**
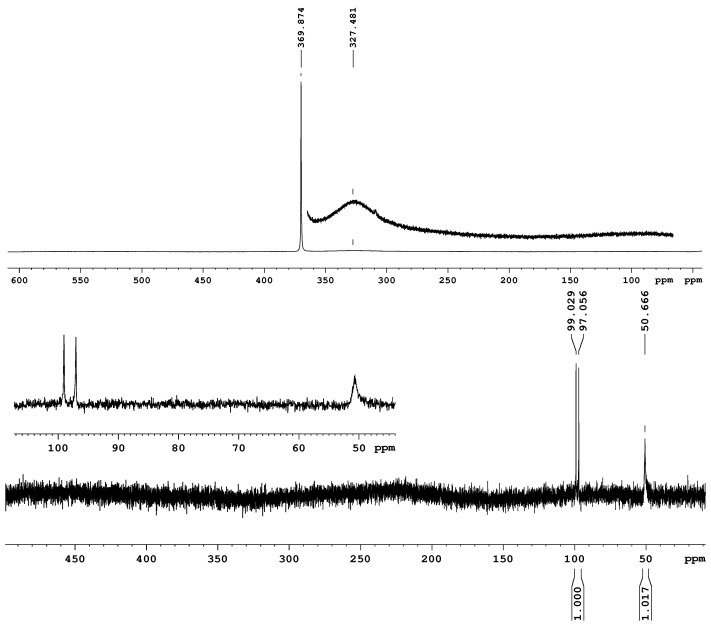
^14^N and ^15^N NMR spectra of sample **6**.

N-NMR study failed to simultaneously yield information about all the particles present in the solution: the ^14^N NMR spectrum showed signals relating only to the dinitramide anion (Figure 3). The position of the signals is consistent with findings previously obtained for other compounds. However, the position of the signals cannot confirm whether there is a coordination of dinitramide with the metal ion; the signal position differs too little from that of the free anion. 

**Figure 4:**
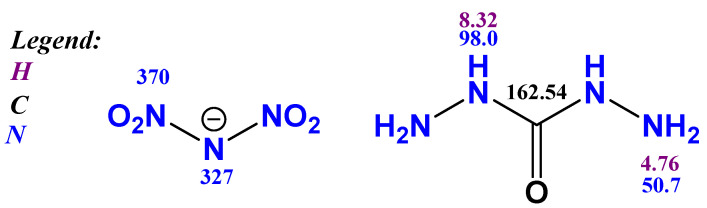
Signal assignment for sample **6**.

In contrast, the ^15^N NMR spectrum (Figure 3) showed two signals from **1**, whereas the dinitramide anion did not manifest itself in the spectrum. The signal near 98 ppm presented as a doublet corresponding to the NH group of the hydrazide moiety with the coupling constant ^1^J_NH_ = 100.3 Hz. The terminal amino group presented as a broadened signal at 51 ppm, with no discernible and separate multiplet lines due to the participation of the hydrogen atoms in chemical exchange and due to N being coordinated with the Zn ion. We failed to acquire a 2D correlation of NH because the relaxation time of the H nuclei was too short. Accordingly, the following signal assignments can be made (Figure 4): ^1^H NMR (500 MHz, DMSO), δ 4.76 (br.s, 2H, NH_2_), 8.32 (br.s, 1H, NH); ^13^C NMR (126 MHz, DMSO), δ 162.54 (CO); ^15^N NMR (51 MHz, HCONH_2_), δ 50.7 (NH_2_), 98.0 (NH); ^14^N NMR (36 MHz, HCONH_2_), δ 327 (N^−^), 370 (NO_2_).

Therefore, it can be concluded that sample **6** contains both the DN anion and carbohydrazide, although it was not possible to establish their ratio. A mutual influence between the DN anion and CHZ is reflected by the overlapped absorption bands of nitro and amino groups in the IR spectrum of **6**. For instance, the absorption band at 1528 cm^−1^ was attributed to NO_2_ vibrations in DN shifted to 1536 cm^−1^, whereas 1539 cm^−1^ was attributed to NH_2_ bending in CHZ shifted to 1605 cm^−1^ (Table 1). 

Mass spectrometry of compound **6** (Supplementary Materials) showed that the spectrum contained no ion of complex **6**. In the spectrum itself, it was possible to detect only ions relating to CHZ, M^+^: 90, 74, 59, 44, and 32 (100%), in agreement with the mass spectrum of carbohydrazide from the database. 

**Scheme 3:**
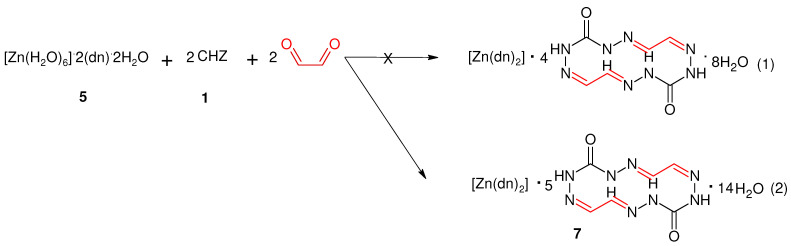
One-pot synthesis of **7** from **5**, **1**, and glyoxal.

In the one-pot synthesis, the aqueous solutions of compounds **5** and **1** and 40% glyoxal solution were mixed in a reactor in series (Scheme 3). The reaction mixture turned a beige color within 10–15 min, and precipitation was observed. 

The reaction was expected to proceed via direction 1, similarly to the reaction between [Ni(dn)_2_] and CHZ and glyoxal [29], but the analysis for Zn and elemental analysis for C, H, and N indicated the production of adduct **7** to be produced (reaction 2).

The resultant sediment was insoluble in water and organic solvents. Therefore, the only available identification method was IR spectroscopy, with confirmation by elemental analysis. 

The aqueous solution of complex **6** is stable under storage conditions, with no decomposition of the target product observed. Adding the aqueous glyoxal solution to complex **6** caused a yellow sediment to precipitate. 

**Scheme 4:**
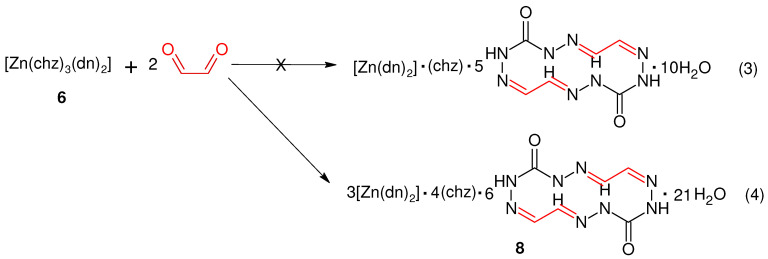
The expected reaction between **6** and glyoxal in a ratio of 1:2.

It was anticipated that compound **6** would react with glyoxal in a ratio of 1:2 to furnish an adduct via reaction direction 3 in Scheme 4 and that one CHZ molecule would remain with the zinc cation. Because the resultant sediment appeared to be insoluble in water and organic solvents, the main identification method was IR spectroscopy with elemental analysis. We were able to partially obtain results by mass spectroscopy and NMR spectroscopy.

Mass spectrometry of compound **8** (Supplementary Materials) showed the complex **8** ion to be absent in the spectrum. In the spectrum itself, we were able to detect ions relating to CHZ, M^+^: 90, 74, 59, 44, and 32 (100%), as well as an ion with *m*/*z* = 224, presumably of C_6_H_8_N_8_O_2_, which is consistent with the composition of the macrocyclic compound. The weight was measured and confirmed for that composition. Furthermore, an ion with *m*/*z* = 415 was detected, presumably related to the [Zn(chz)·C_6_H_8_N_8_O_2_·2H_2_O] fragment from the major ion of compound **8**.

Therefore, it can be concluded that the synthesized adduct **8** structurally contains carbohydrazide **1** and macrocycle **2**.

The samples of compounds **7** and **8** exhibit an extremely low solubility of ~1 mg/0.6 mL DMSO. The resultant yellow solutions had extremely low concentrations, and even the spectrum from the ^13^C nucleus (Figure S18 Supplementary Materials) did not yield any meaningful information (6.5 h accumulation time).

The presence of the DN anion in compounds **6**–**8** was shown by IR spectroscopy through vibrational bands of NO_2_ linkages (1519–1523 cm^−1^, 1179–1187 cm^−1^) using the comparison base of [Zn(dn)_2_] and [Ni(chz)_3_(dn)_2_] [18]. The IR spectra of the obtained sediments **7**–**8** were comparatively analyzed to show that the bands of the functional groups belong to both monocycle **2** [26] and compound [Zn(dn)_2_] (Table 1).

As shown in Table 1, the shifts in absorption bands of the NO_2_ stretching vibrations are well correlated, that is: 

(*ν*_as_*)*: 1537 cm^−1^ ([Ni(chz)_3_(dn)_2_]) = 1537 cm^−1^ ([Zn(dn)_2_]) >1536 (**6**) > 1523 (**8**) > 1519 cm^−1^ (**7**);

(*ν*_s_): 1187 cm^−1^ (**7**) >1180 cm^−1^ (**8**) >1178 cm^−1^ (**6**) >1177 cm^−1^ ([Zn(dn)_2_]) = 1177 cm^−1^ ([Ni(chz)_3_(dn)_2_]).

A tendency was observed of no shifts in absorption bands for the *ν*(C=O) group engaging in coordination with the metal, that is: 1644 cm^−1^ ([Ni(chz)_3_(dn)_2_]) = 1644 cm^−1^ (**6**).

It is worth noting that the IR spectrum of **8** differs greatly from that of compound **7** in the region of *δ*NH bending vibrations and has a high-intensity absorption band (1616 cm^−1^) that is absent in the spectrum of compound **7**. This absorption band can be attributed to the CHZ found in the structure of adduct **8**, as confirmed by mass spectrometry. 

Thus, the study results show that the synthesized compounds **6**–**8** structurally contain the DN anion, whereas adduct **8** additionally comprises monocycle **2** and CHZ. Analysis of the zinc cation showed it to be present in all of the samples. 

DSC studies demonstrated that compound **6** has a melting temperature of 179 °C (or phase transition) and a peak of decomposition of 222 °C (Table 2). 

As shown in Table 2, adducts **7** and **8** have three decomposition stages. Stage I in the DSC spectrum is characterized by an intense decomposition peak at 97 °C with a 90 J/g heat release for compound **8** and at 101 °C with a 99 J/g heat release for compound **7**. However, the weight loss is 12.4602% for **7** and 11.6526% for **8** (Supplementary Materials, TGA: Figures S8 and S10), which is consistent with the calculated removal of 11 mol H_2_O for **7** and 19 mol H_2_O for **8**. The second stage in the DSC spectrum has an intense decomposition peak at 212 °C with a 162 J/g heat release for compound **8** and at 214 °C with a 81 J/g heat release for compound **7**. The weight loss is 29.5789 % for **8** and 16.9641 % for **7**. The third stage in the DSC spectrum has an intense decomposition peak at 266 °C with a 301 J/g heat release for compound **8** and at 274 °C with a 381 J/g heat release for **7**. The weight loss is 31.3381 % for compound **8** and 45.9226% (including the 2nd stage) for **7**. The solid residue is 27. 4304% for compound **8** and 23.653% for **7**.

Based on the comparative data on the decomposition temperatures of **6** and **10**, we the peaks at 214 °C (**7**) and 212 °C (**8**) can be assigned to the decomposition of the constituent part of the DN anion. The third stage of the decomposition of **7** (274 °C) and **8** (266 °C) is characterized by a decomposition of macrocycle **2** (it decomposes at 298 °C) found in their structures. 

Adducts **7** and **8** have the lowest sensitivity to mechanical stimuli among the tested compounds (**6** and **10**–**11**), as shown in Table 3, potentially reducing danger when handling them. However, given that the synthesized complex **6** has a higher cumulative heat release per unit weight during the first and second decomposition stages of 858 J/g (1000 J/g–142 J/g) compared to 561 J/g and 553 J/g for adducts **7** and **8** (Table 2), respectively, we performed the subsequent tests with complex **6**.

### 3.2. Testing of Compound **6** for the Effect on Pyrotechnic Composition Burning Rate

We carried out comparative trials to evaluate the effect **6** on the burning rate of the pyrotechnic mixtures. Two formulations differing in burning rate were chosen as model compositions to identify the potential effect (both catalytic and inhibitory effects). 

The first model formulation was potassium perchlorate blended with aluminum in a ratio of 70/30. The burning rate of this mixture is not fast (1.8058 mm/s) with no additives. 

The second model formulation was a mixture of 48/52% potassium nitrate/zirconium. This mixture has a burning rate of 34.6617 mm/s without additives. 

To determine how compound **6** affects the pyrotechnic mixture burning rate, compound **6** was tested in small quantities: 0.5%, 1.0%, and 1.5% on top of 100% of the weight. 

**Figure 5:**
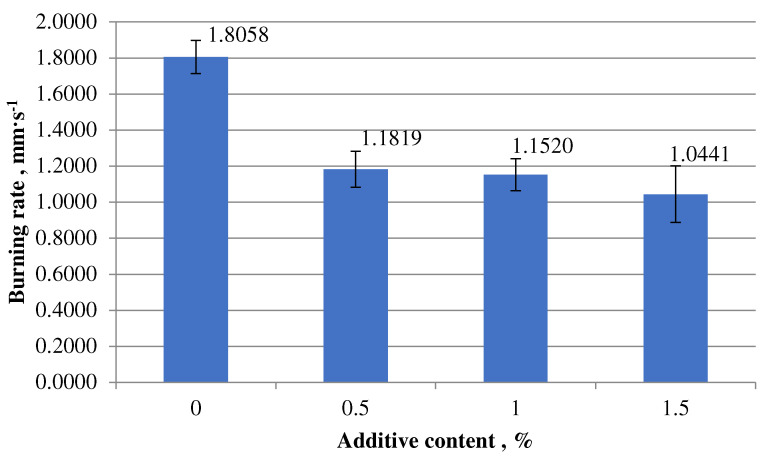
Influence of **6** on the burning rate of mixed potassium perchlorate/aluminum.

**Figure 6:**
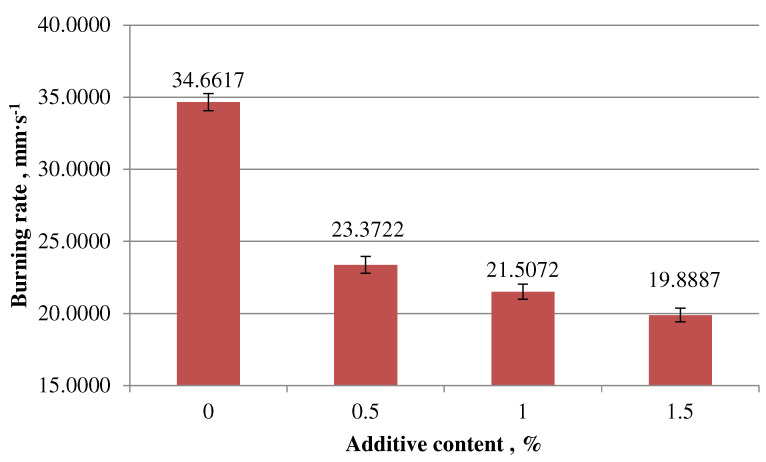
Effect of **6** on the burning rate of mixed potassium nitrate/zirconium.

The evaluation results of the effect of compound **6** on the pyrotechnic compositions are illustrated in Figure 5 and Figure 6. 

Figure 5 demonstrates that the KClO_4_/Al burning rate declines as the content of **6** in the system increases. The burning rate diminishes from 1.0858 mm/s to 1.0441 mm/s.

Figure 6 demonstrates that an inhibitory effect is also observed for the KNO_3_/Zr system. The burning rate also decreases as the additive content is increased. The burning rate diminishes from 34.6617 mm/s to 19.8887 mm/s.

Comparative data on the inhibitory activity as a function of the additive content are presented in Table 4. 

The data presented in Table 4 demonstrate that compound **6** exhibits 42% inhibitory activity towards the KClO_4_/Al formulation, with up to 42% inhibition in the KNO_3_/Zr formulation when the additive content is between 0.5% and 1.5%. The reduction in the burning rate of the model pyrotechnic mixtures is of practical importance, although it cannot be confirmed at this point as to which factor is the determinant of the burning-rate reduction for fast-burning and slow-burning pyrotechnic compositions. The answer to this question requires additional studies to identify the burning-rate inhibition mechanism.

## 4. Conclusions

1. A coordination compound, tris(carbohydrazide-N,O)zinc(II)dinitramide, was synthesized herein through a reaction between zinc dinitramide and carbohydrazide. The structure of the compound was identified by physicochemical analytical methods and proven by X-ray diffraction.
2. The reaction of zinc dinitramide with carbohydrazide and glyoxal via one-pot synthesis furnished the respective adducts, depending on the molar ratio of the starting reactants. Mass spectroscopy showed the adduct to structurally contain carbohydrazide and macrocyclic (1,2,4,5,8,9,11,12-octaazacyclotetradeca-5,7,12,14- tetraene-3,7-dione). The resultant adducts comprising zinc dinitramide are not soluble in water or organic solvents.
3. The coordination compound, tris(carbohydrazide-N,O)zinc(II)dinitramic acid, was tested herein as a modifier of the burning rate of pyrotechnic mixtures. The zinc salt of dinitramide was discovered to reduce the burning rates of KClO_4_/Al and KNO_3_/Zr and can be employed as an inhibitor of the burning rate of pressed pyrotechnic charges.

## Figures and Tables

**Table 1:** IR frequencies of **1**, **2**, **6**–**8**, and **9**.

Comp.	Frequency, cm^−1^						
**6**	1644 s.	1605 s.	1536 v.s.	1430 s., 1344 m.	1204 v.s., 1178 v.s.	1088, 1031, 1022	828, 762, 731
**9**	1644 v.s.	1604, 1542 s.,	1537 v.s.	1427, 1344 m.	1202 v.s., 1177 v.s.	1096, 1022 s.	828, 762, 732
**7**	1693 v.s.	Abs.	1519 v.s.	1443 w., 1361 m.	1187 s.,	1020	826, 762, 740
**8**	1665 s.	1616 s.	1523 v.s.	1444 m., 1354 m.	1180 s.	1014	826, 756
**5**	Abs.	Abs.	1537 v.s.	1432 s., 1344 m.	1205 v.s., 1177 v.s.	1031, 1023 s. doublet	827, 762, 732
**1**	1639 s.	1539	Abs.	1455, 1344 m., 1320	Abs.	1048	Abs.
**2** [26]	1666 s.	1587 s.	Abs.	1308	Abs.	1008 w.	Abs.
Functional groups	*υ* C=O	*δ* NH	*ν* N=N-O	*δ* NH	*ν* N=N-O	N-N, N-N-N	NO_2_

**9**—[Ni(chz)_3_(dn)_2_] [18]. Intensities: *υ*—stretching; *δ*—bending; s.—strong; Abs.—absent; sh.—shoulder; w.—weak; m.—medium; v.—very. Red color indicates the similarity between the absorption bands of **6**, **7**, and **8** and the NO_2_ vibrational frequencies of the known compounds (**5** and **9**) bearing the DN anion.

**Table 2:** Decomposition temperatures of **1**, **2**, **6**–**8**, and **10** as per DSC data.

Comp.	Stage I				Stage II				Stage III			
	Onset, °C	Peak, °C	End Set, °C	Specific Heat Release, J/g	On Set, °C	Peak, °C	End Set, °C	Specific Heat Release, J/g	On Set, °C	Peak, °C	End Set, °C	Specific Heat Release, J/g
6	177	179 *	180	−142	210	222	237	1000	-	-	-	-
10	31	37 *	42	−31	184	216	218	1307	255	261 *	262	-9
7	43	101	109	99	188	214	236	81	265	274	283	381
8	61	97	145	90	188	212	236	162	255	266	280	301
2 [26]	-	-	-	-	-	-	-	-	288	298	291	1056
1	156	157 *	160	−321	-	-	-	-	-	-	-	-

* Melting point. **10**—dinitramide carbohydrazide [18].

**Table 3:** Physicochemical properties of compounds **6**, **7**, and **8**.

Comp.	Color	Density, g/cm^3^	T dec., °C	Sensitivity		Zn Content, %
				Impact (10 kg), mm	Friction, kgf/cm^2^	
**6**	Yellow hue	1.9216	222	<50	1400	12.04
**8**	Dark yellow	1.7283	266	70	3600	6.75
**7**	Dark yellow	1.6237	274	70	3200	3.99
**10**	–	–	–	<50	2500	–
**11**	–	–	–	<50	2400	–

**10**—dinitramide carbohydrazide [18]; **11**—bis-dinitramide carbohydrazide [18].

**Table 4:** Burning rate as a function of the content of **6** in the pyrotechnic mixtures used in the present study.

Additive	∇u (KClO_4_/Al), %	∇u (KNO_3_/Zr), %
0.5% 6	−34.55	−32.57
1.0% 6	−36.21	−37.95
1.5% 6	−42.18	−42.62

## Data Availability

Not applicable.

## Supplementary Materials

The following supporting information can be downloaded at https://www.mdpi.com/article/10.3390/ma16010070/s1, containing IR spectra of **6**, **7**, and **8**; ^1^H and ^13^C NMR spectra of **6**, **7**, and **8**; MS analysis of **6**, **7**, and **8**; X-ray structure of **6**; and DSC and TGA of **6**, **7**, and **8**.
