# Supplementary material for: Adducts of the Zinc Salt of Dinitramic Acid
Source: Materials (Basel). 2022 Dec 21;16(1):70. doi: 10.3390/ma16010070 (PMC9821768; doi:10.3390/ma16010070)
Supplement: Supplementary file 1 [file materials-16-00070-s001.zip › materials-2117592-supplementary.pdf]

Supplementary Materials

Adducts of the Zinc Salt of Dinitramic Acid

Sergey G. Il'yasov [1,*], Vera S. Glukhacheva [1], Dmitri S. Il'yasov [1], Egor E. Zhukov [1], Ilia V. Eltsov [2], Andrey A. Nefedov [2,3] and Yuri V. Gatilov [3]

[1] Institute for Problems of Chemical and Energetic Technologies, Siberian Branch of the Russian Academy of Sciences (IPCET SB RAS), 659322 Biysk, Russia
[2] Department of Organic Chemistry, Faculty of Natural Sciences, Novosibirsk State University, 630090 Novosibirsk, Russia
[3] Department of Physical Organic Chemistry (OPOC), Vorozhtsov Novosibirsk Institute of Organic Chemistry, Siberian Branch of the Russian Academy of Sciences (NIOCh SB RAS), 630090 Novosibirsk, Russia
* Correspondence: ilysow@ipcet.ru

Table of Contents

IR spectra of **6, 7, 8,** S1-S3

X-ray structure of **6** S4-S6

DSC and TGA of **6, 7, 8** S7-S9

MS analysis of **6, 7, 8** S10-S13

^{1}H and ^{13}C NMR spectra of **6**,**7**,**8** in DMSO-d6 S14-S18

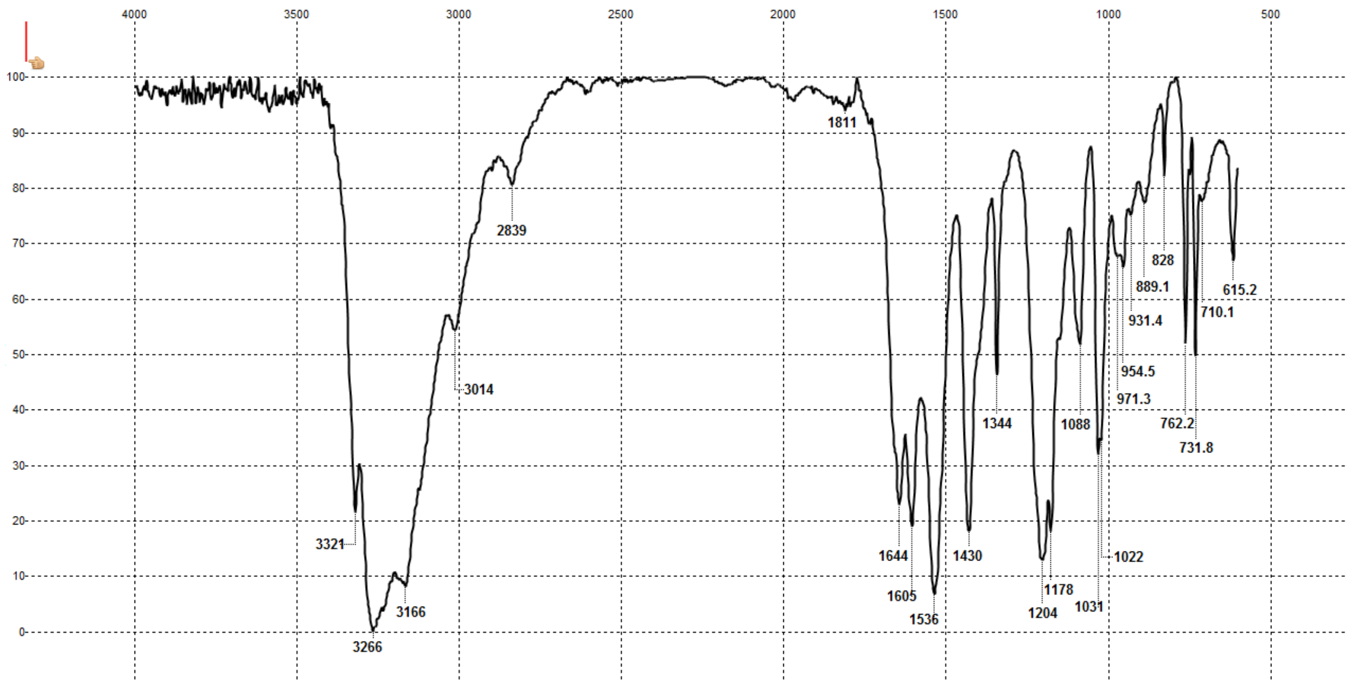

Figure S1. IR spectrum of **6**

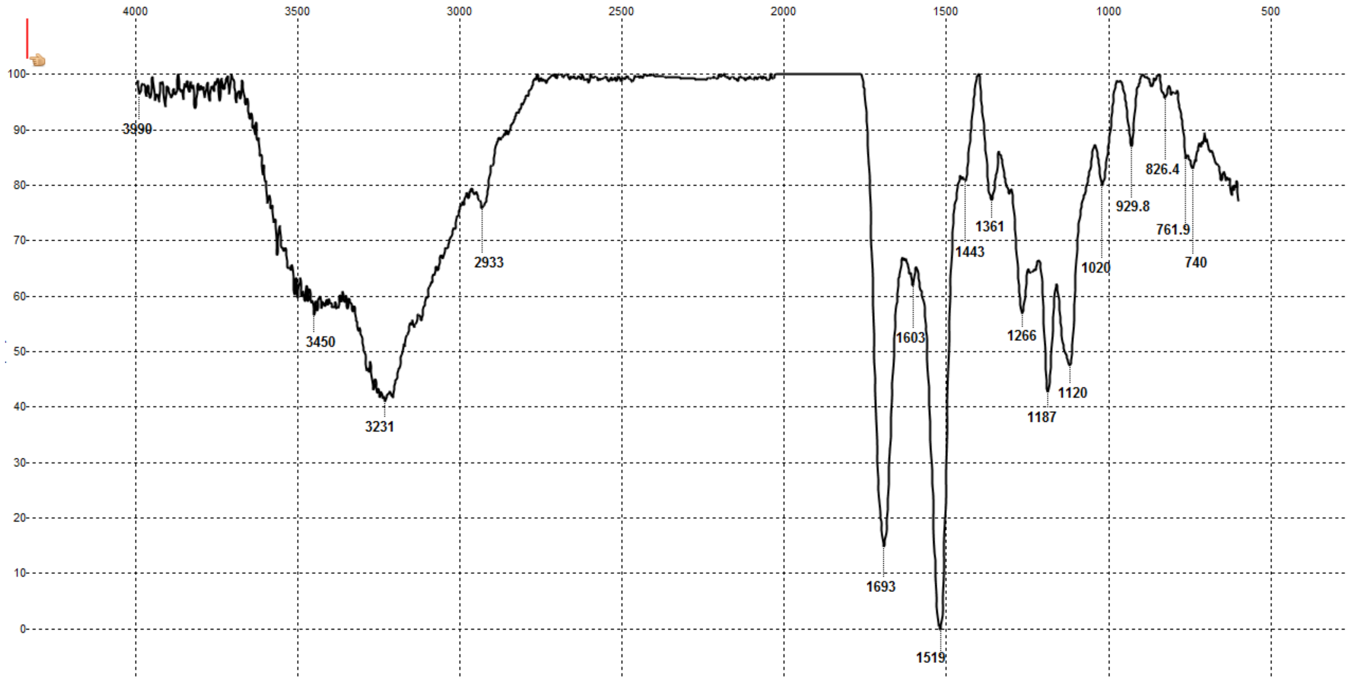

Figure S2. IR spectrum of **7**

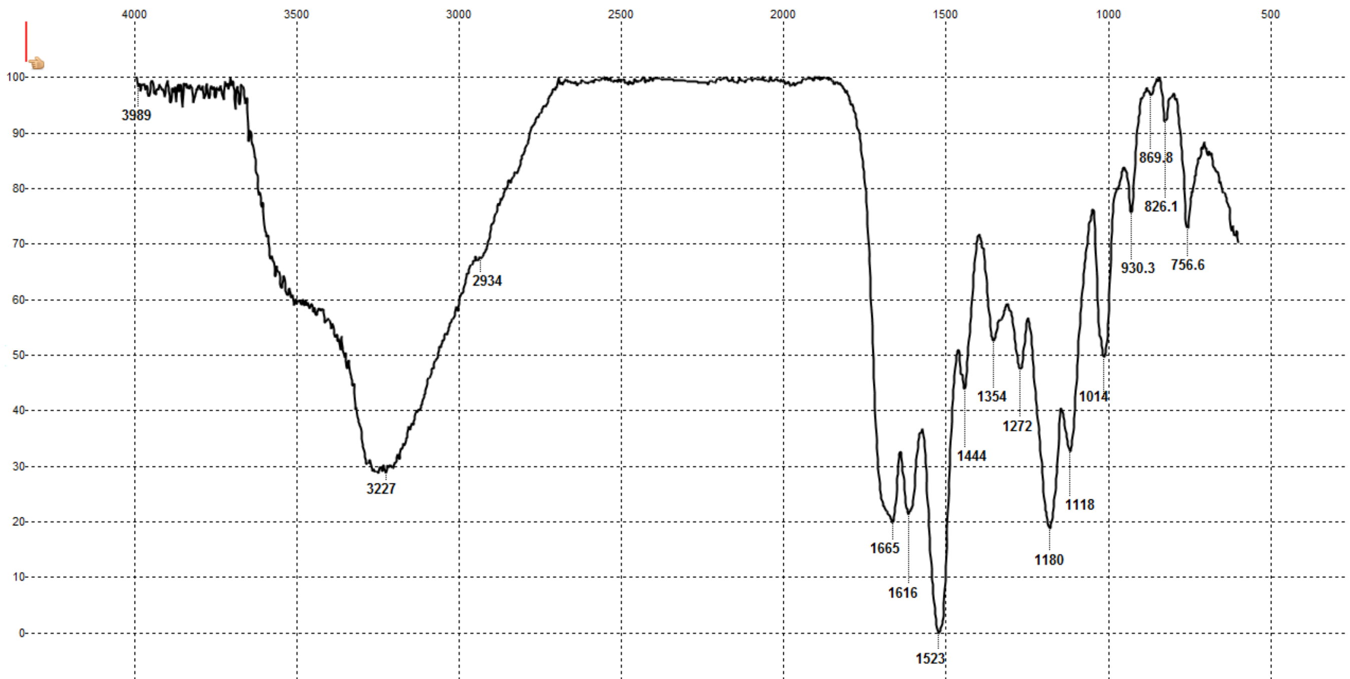

Figure S3. IR spectrum of **8**

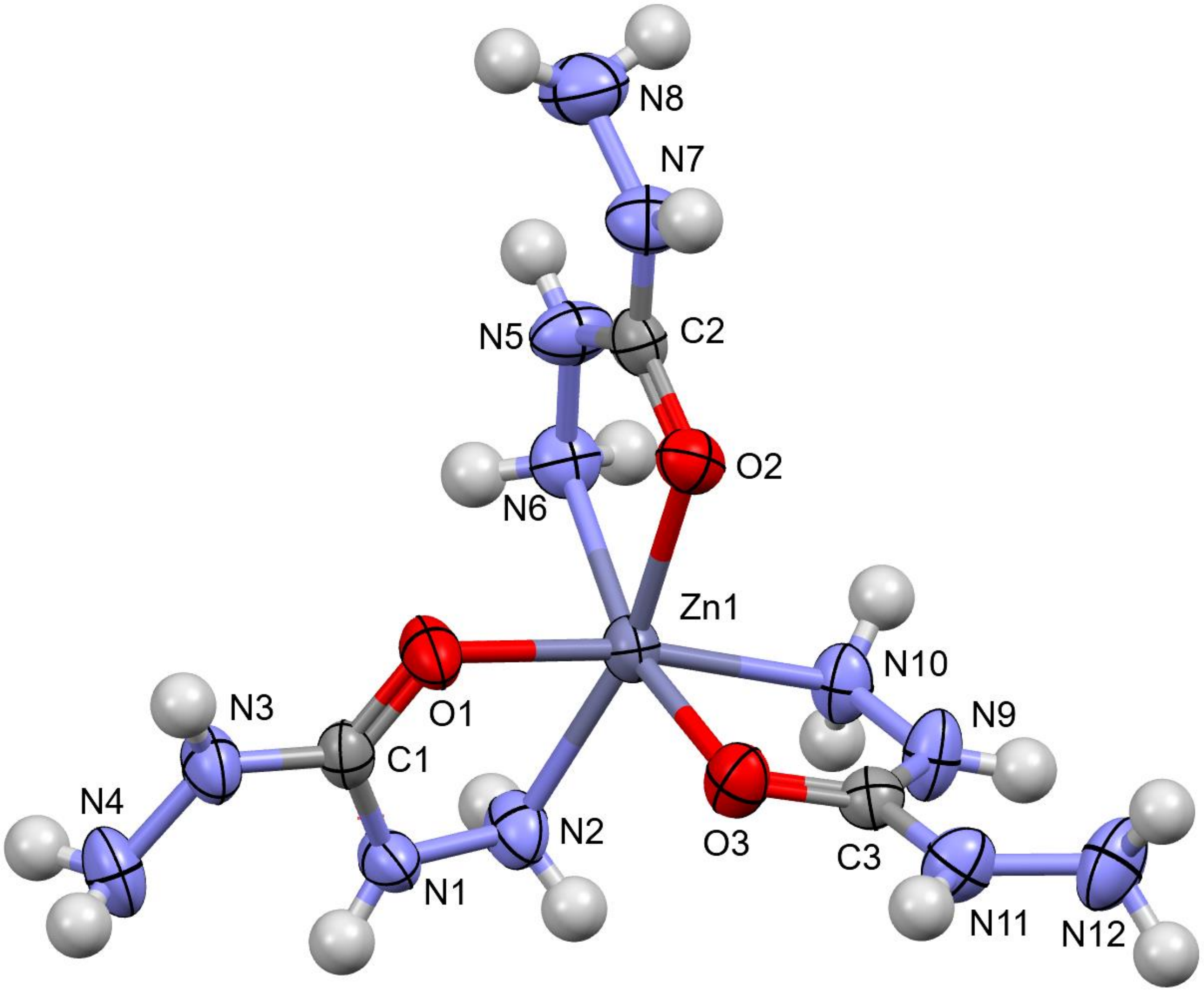

Figure S4. X-ray structure of **6**. Thermal ellipsoids are shown at 50% probability. Only one cation of two independent is shown.

Table S1. Crystal data and structure refinement for **6**

Empirical formula	$C_3H_{18}N_{12}ZnO_3$, $2(N_3O_4)$, $0.5(H_2O)$
Formula weight	556.7
Temperature	296(2)
Wavelength	0.71073
Crystal system	Monoclinic
Space group	$P2_1/c$
Unit cell dimensions	a = 12.993(3) Å α = 90º
	b = 8.875(2) Å β = 92.739(6)º
	c = 35.138(9) Å γ = 90º
Volume	4047.2(17) Å^3
Z	8
Density (calculated)	1.827 g/cm^3
Absorption coefficient	1.311 mm^{-1}
F(000)	2280
Crystal size	0.06 x 0.13 x 0.49 mm^3
Theta range for data collection	1.57 to 26.03°
Index ranges	-16<= h <=15, -10<= k <=10, -43<= l <=43
Reflections collected	46823
Reflections independent, R_{int}	7965, 0.0441
Observed Data [I>2σ(I)]	6453
Completeness to theta = 25.03°	1.000
Absorption correction	Semi empirical from equivalents
Max. and min. transmission	0.7453 and 0.6355
Refinement method	Full matrix least squares on F^2

Data / restrains / parameters	7965 / 260 / 659
Goodness-of-fit on F^2	1.003
Final R indices [I>2σ(I)]	R_1 = 0.0595, wR_2 = 0.1544
R indices (all data)	R_1 = 0.0737, wR_2 = 0.1651
Extinction coefficient	n/a
Largest diff. peak and hole	1.687 and - 0.635 e/$Å^3$
CCDC deposition number	2213295

Table S2. Selected bond lengths (Å) of **6**

Zn1-O1	2.093(3)	Zn1-O2	2.142(3)	Zn1-O3	2.081(3)
Zn1-N2	2.158(4)	Zn1-N6	2.178(4)	Zn1-N10	2.147(4)
Zn2-O4	2.109(3)	Zn2-O5	2.073(3)	Zn2-O6	2.116(3)
Zn2-N14	2.180(4)	Zn2-N18	2.183(4)	Zn2-N22	2.151(4)

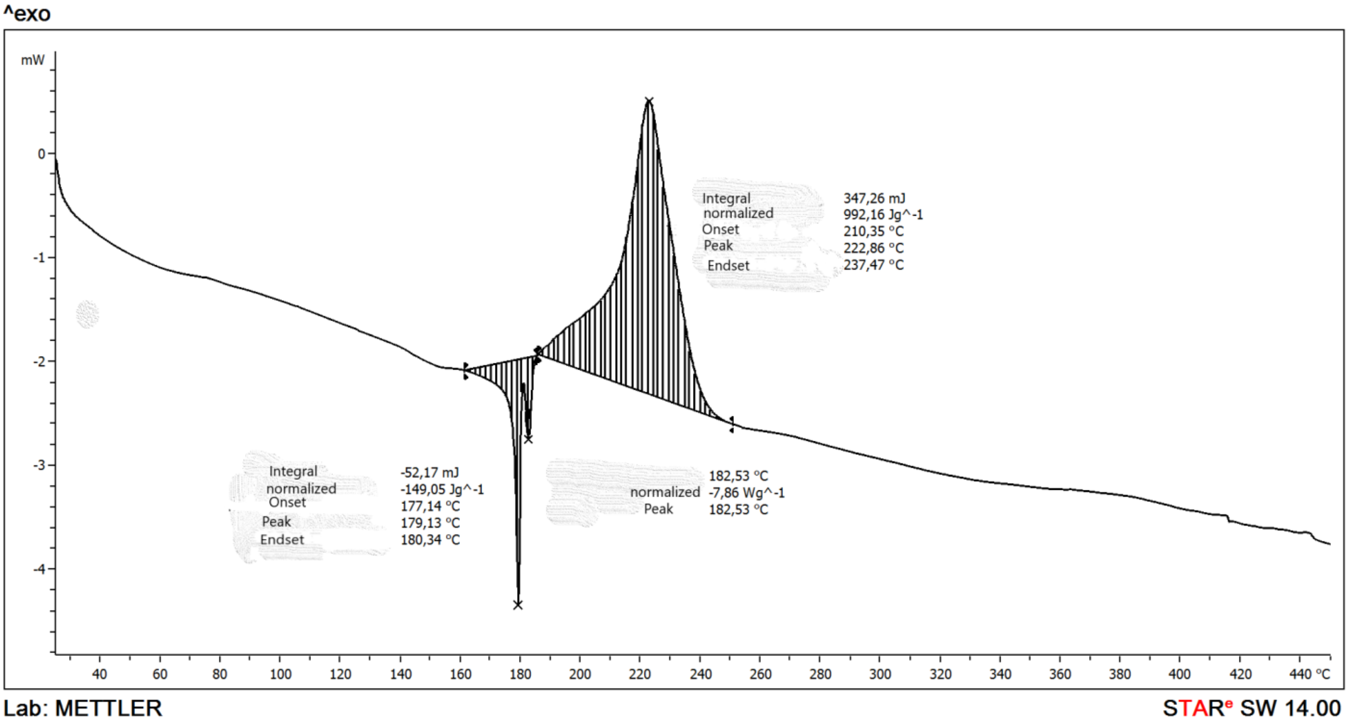

Figure S5. DSC of **6**

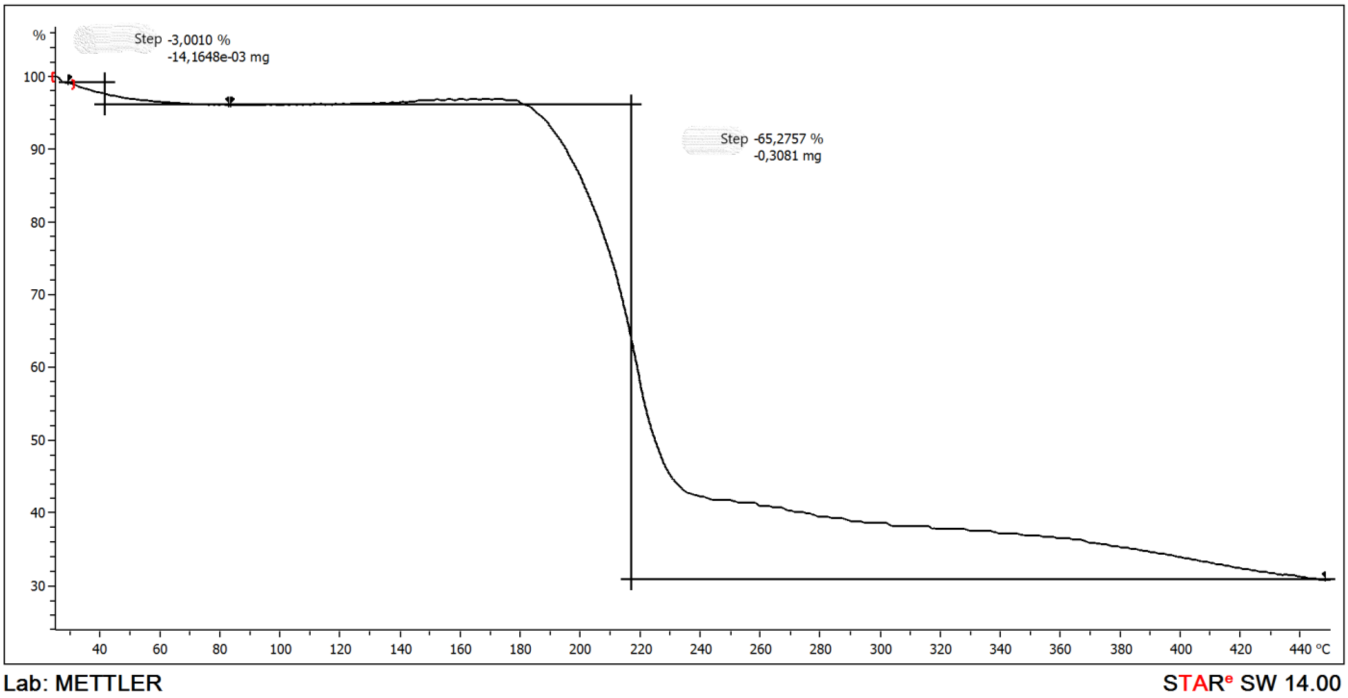

Figure S6. TGA of **6**

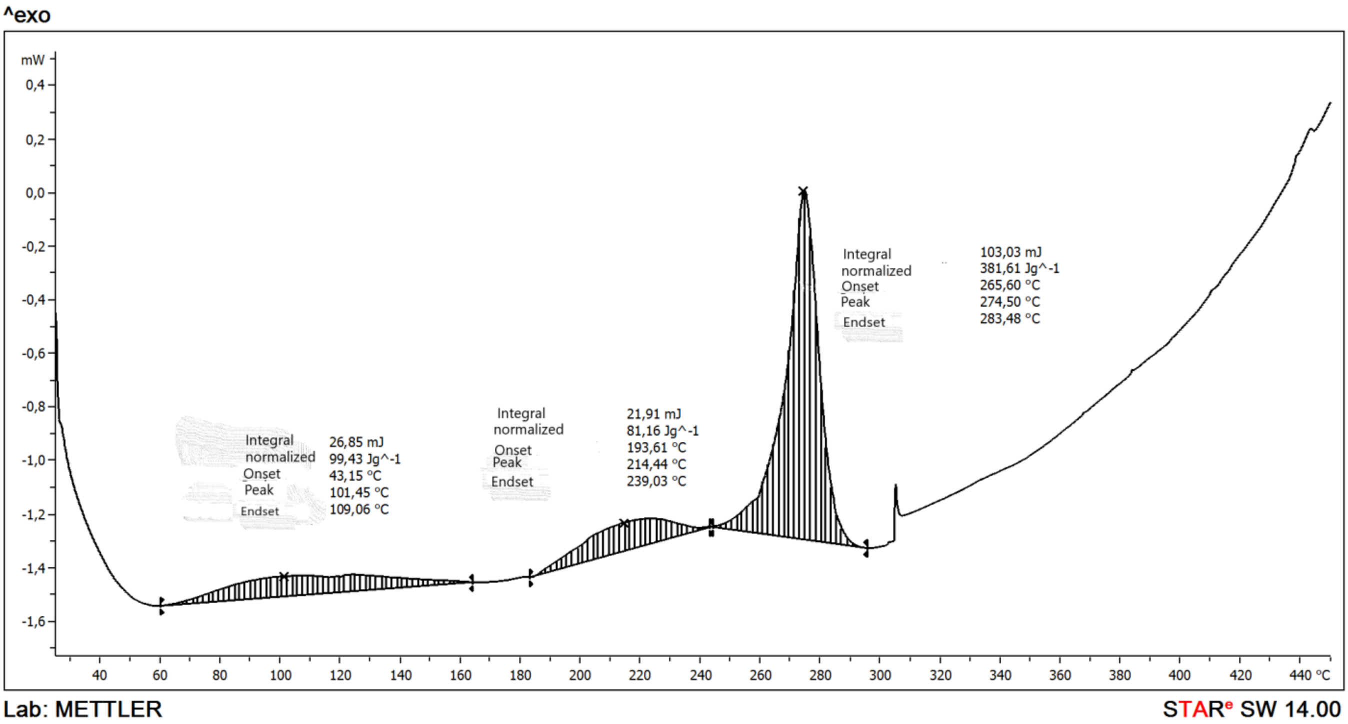

Figure S7. DSC of **7**

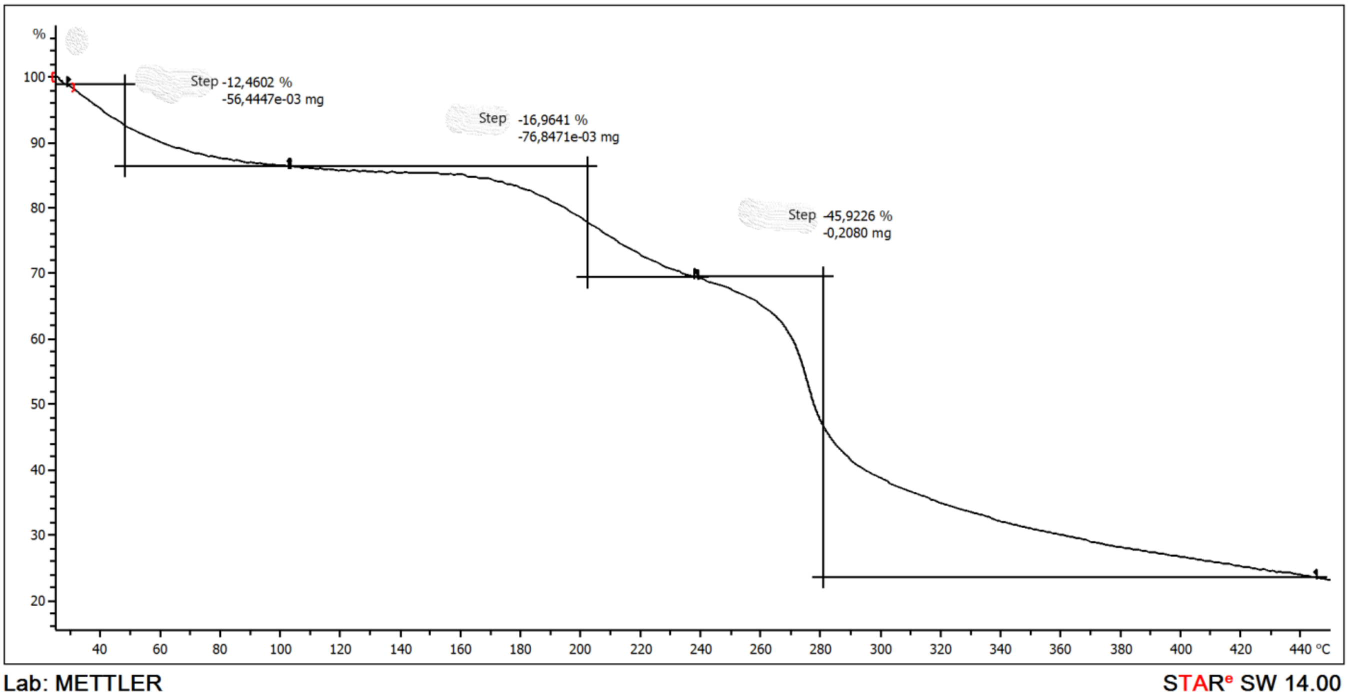

Figure S8. TGA of **7**

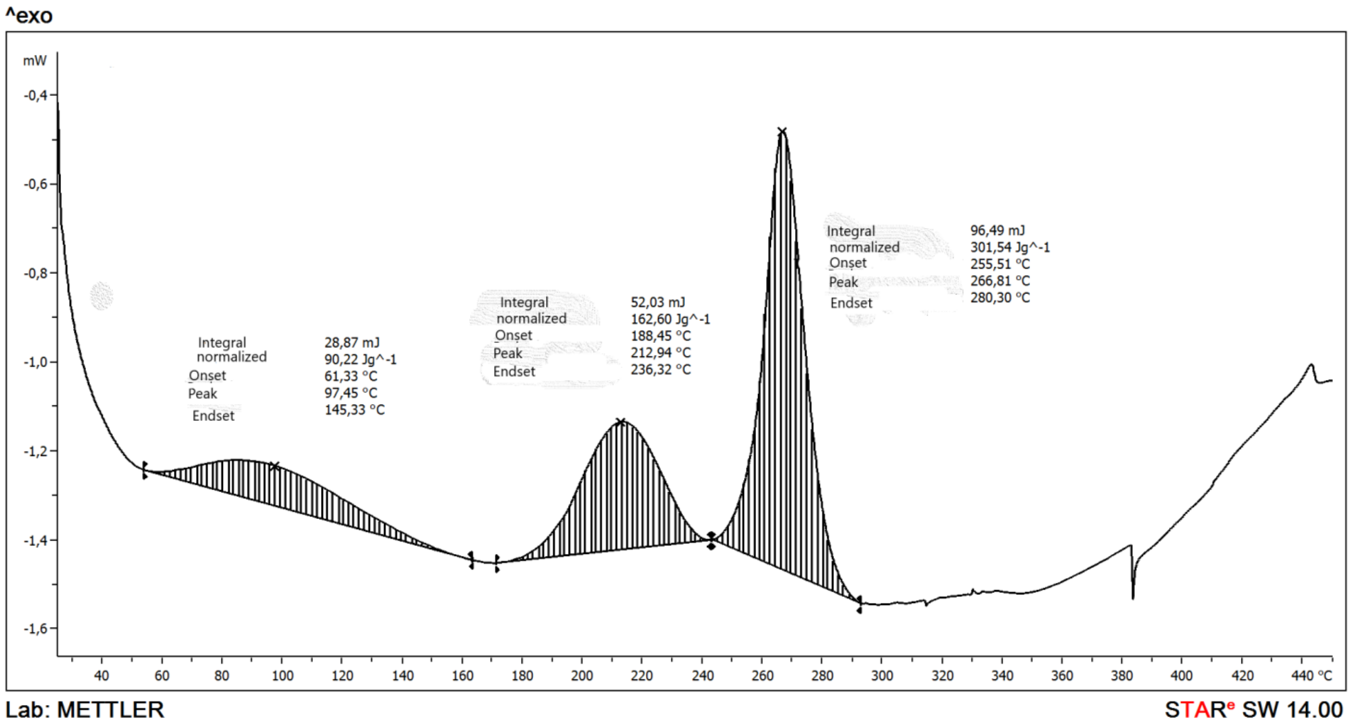

Figure S9. DSC of **8**

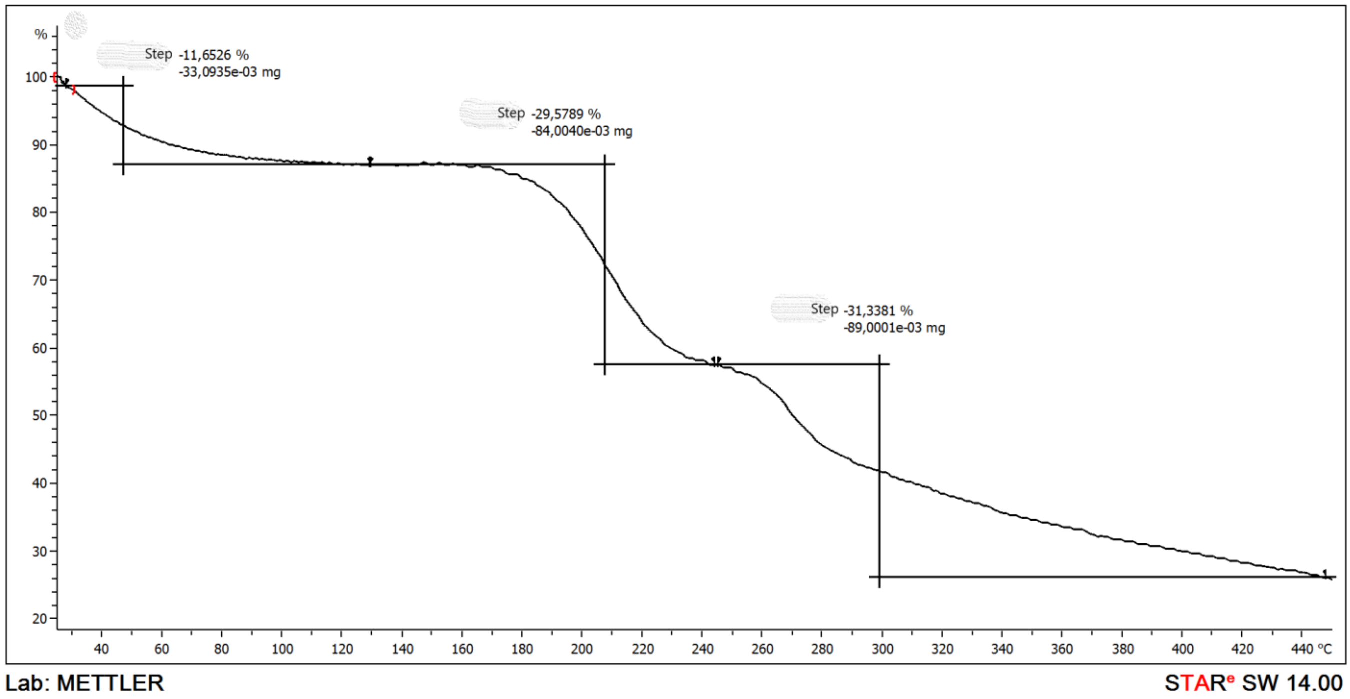

Figure S10. TGA of **8**

Figure S11. MS analysis of **6**

Mass spectrometry and precise measurements of molecular weights were done on a Thermo Electron Double Focusing System (Thermo Electron Corp., USA). The samples contained in metal vials were put into the mass spectrometer by direct inlet system; if necessary, the sample vial can be heated up in the temperature range from 25 to 360°C. The mass spectrometer was operated in the electron ionization mode at electron energy of 70 eV. Measurements of accurate ionic masses were performed with respect to perfluorokerosene (PFK) as internal mass standard.

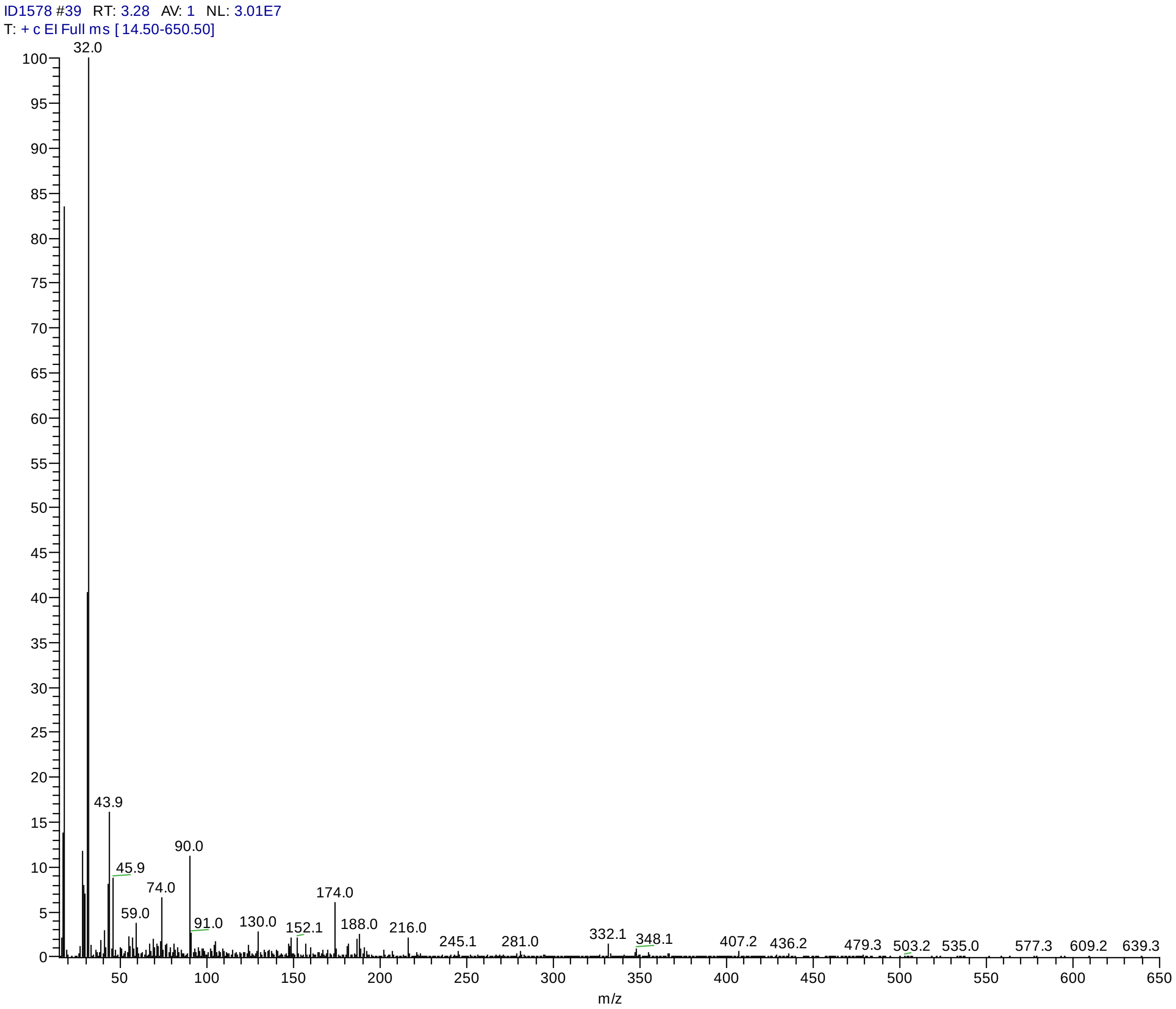

The spectrum contains ions coming from compound соединению (**1**), M+ - 90, 74, 59, 44, 32 (100%), see the mass spectrum from the database:

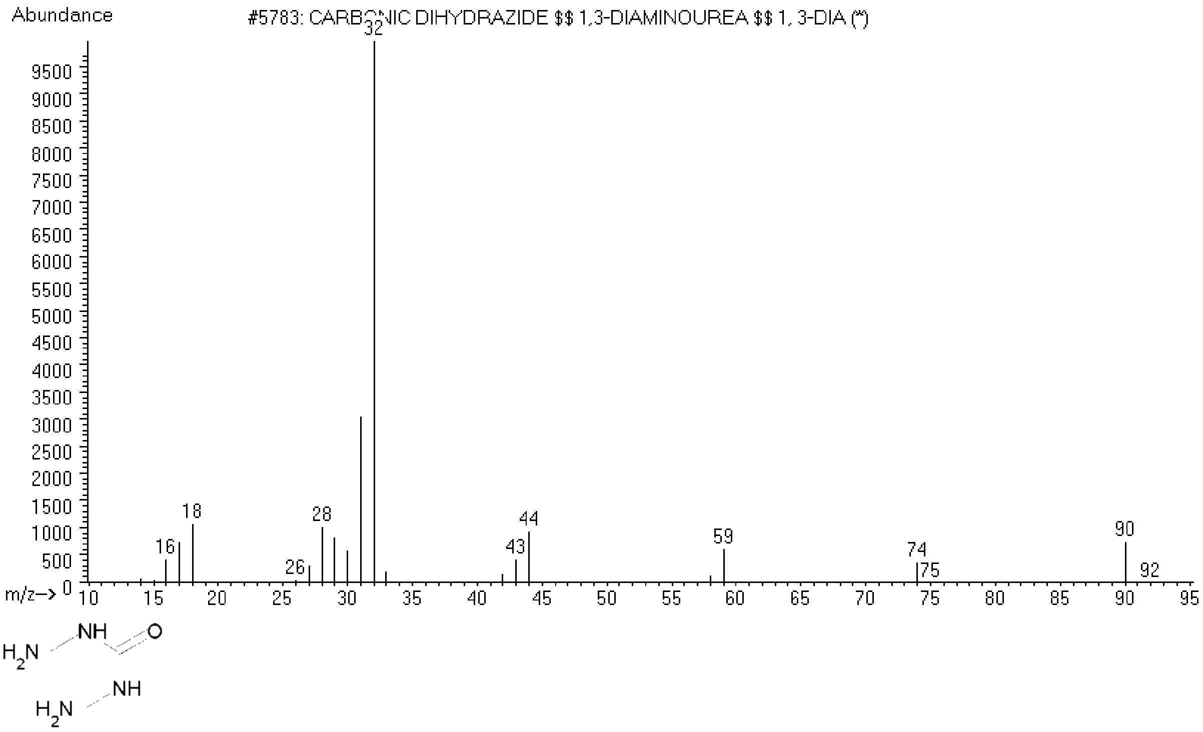

MS for the ions of (a) *m/z* 90 of **1**.

Calculation of elemental compositions

Calculated m/z=90.0537 ($C_1H_6N_4O$)$^{+\cdot}$

Measured m/z=90.0541

	Elemental composition	RDB	Error (ppm)
1	$C_1 H_6 N_4 O$	1.0	4.442

Range of number values of atoms in elements:

Isotope	min	max
**12 C**	**1**	**10**
**1 H**	**1**	**20**
**16 O**	**0**	**6**
**14 N**	**0**	**6**

Figure S12. MS analysis of **8**

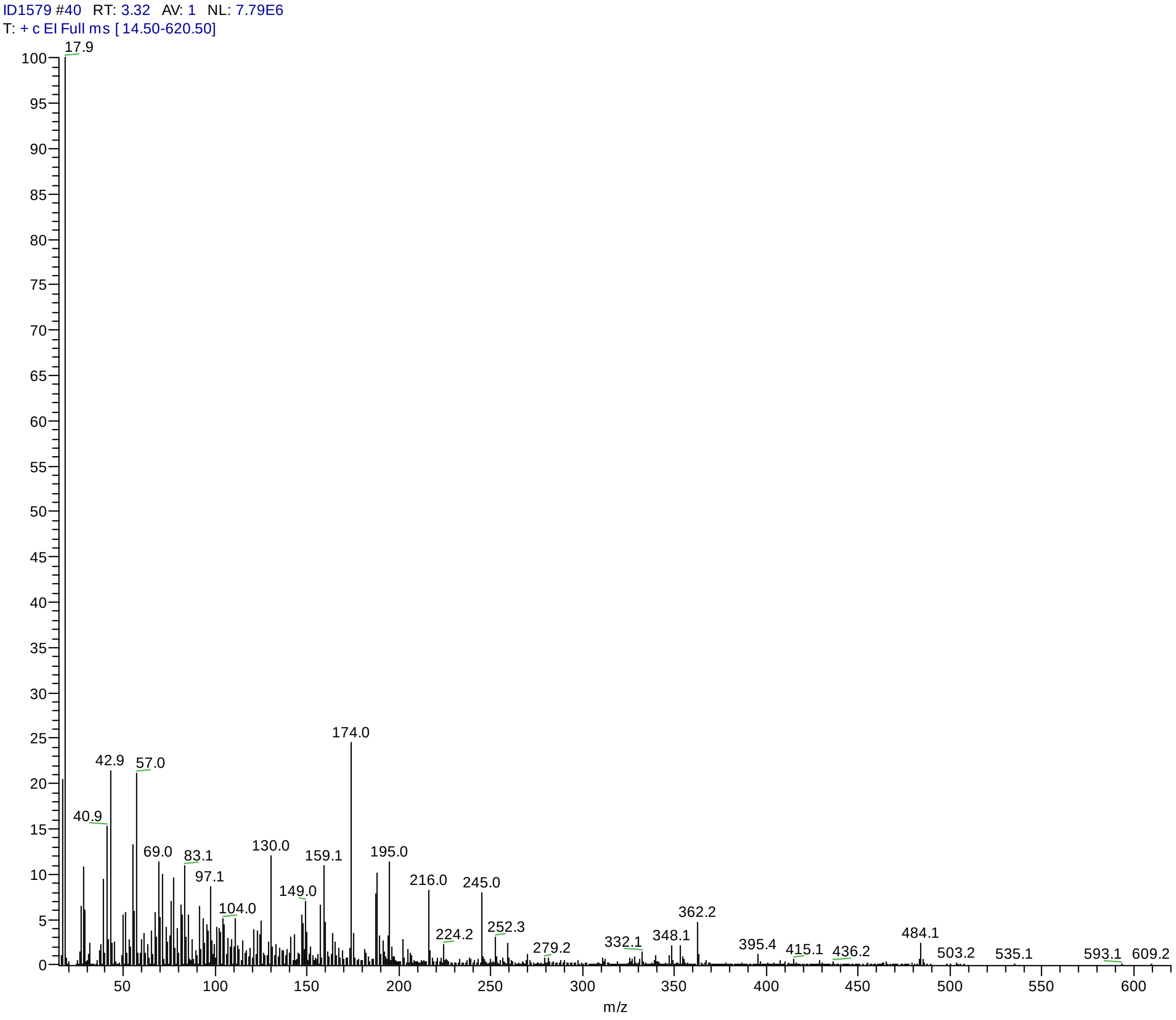

The spectrum contains ions related to carbazide (**1**), M^{+} - 90, 74, 59, 44, 32 (100%), and an ion with m/z=224, presumably $C_6H_8N_8O_2$, corresponding the composition of compound **4**. The weight was measured and confirmed for that composition.

Calculation of elemental compositions

Calculated m/z=224.0765 $(C_6H_8N_8O_2)^{+\cdot}$

Measured m/z=224.0759

Elemental composition		RDB	Error (ppm)
1	$C_6\ H_8\ N_8\ O_2$	7.0	2.678

Range of number values of atoms in elements:

Isotope	min	max
**12 C**	**1**	**10**
**1 H**	**1**	**20**
**16 O**	**1**	**10**
**14 N**	**1**	**10**

Figure S13. MS analysis of **7**

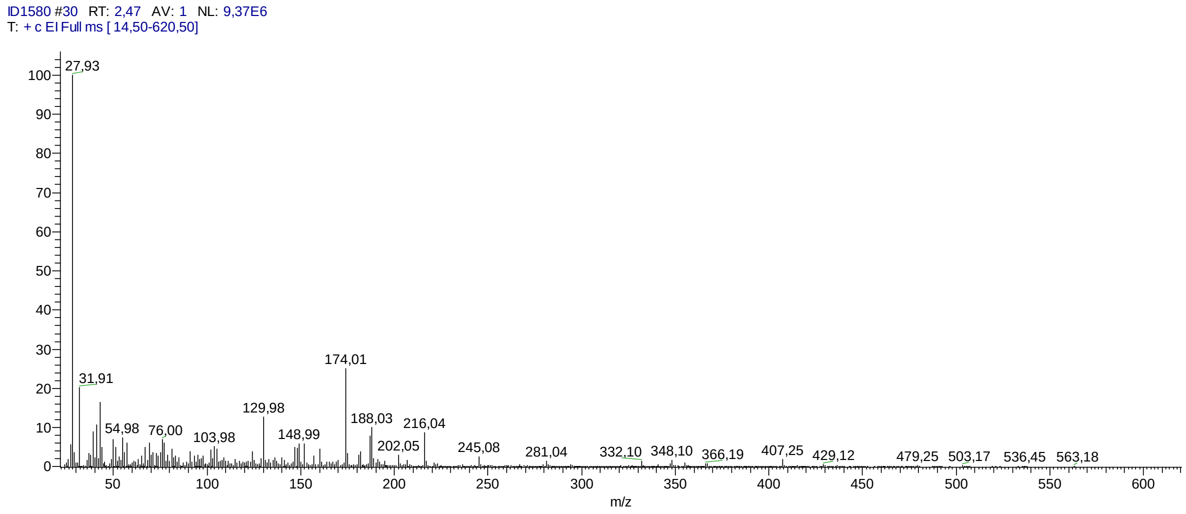

MS for the ions of (a) *m/z* no ion with m/z=224, which was detected and confirmed for the composition, as shown above.

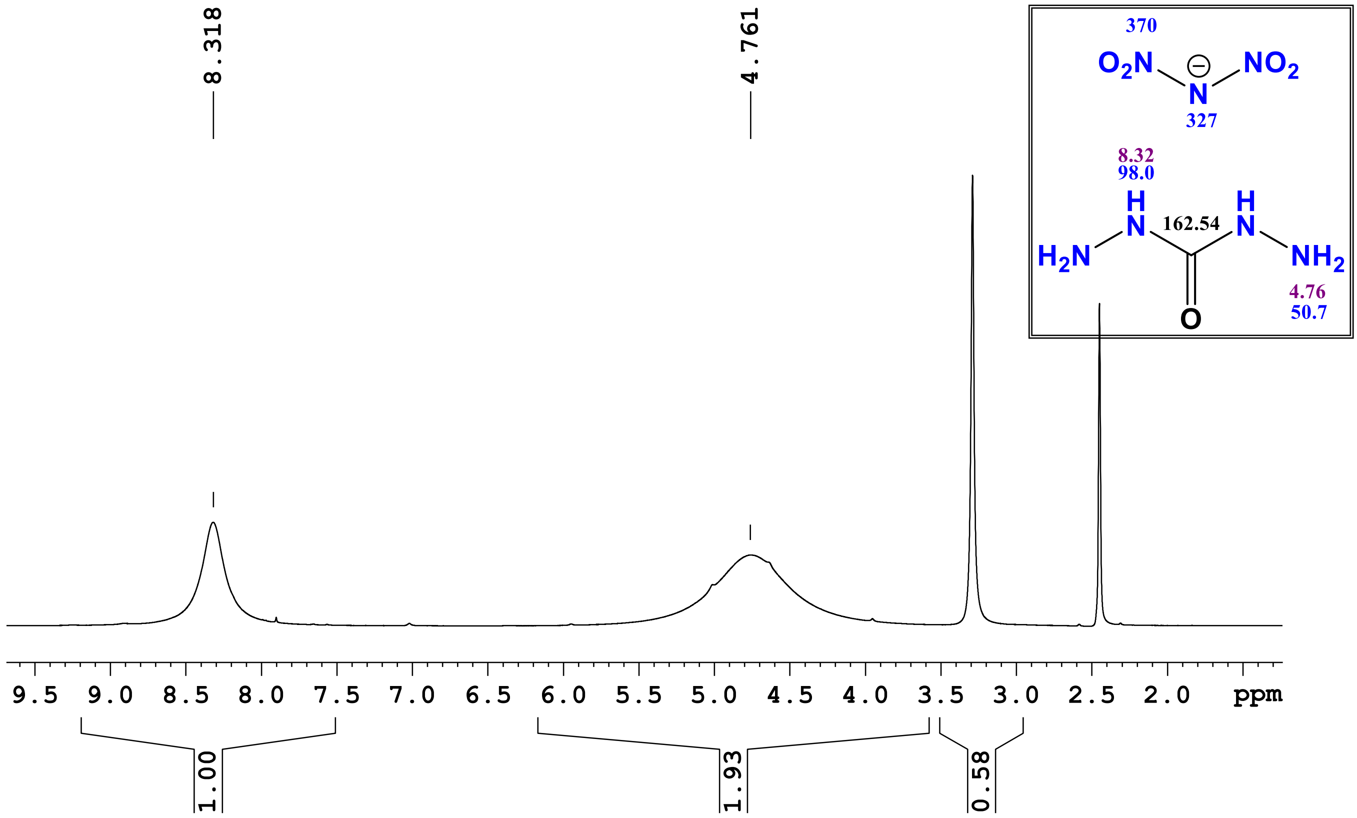

Figure S14. ^{1}H-NMR spectrum of **6**

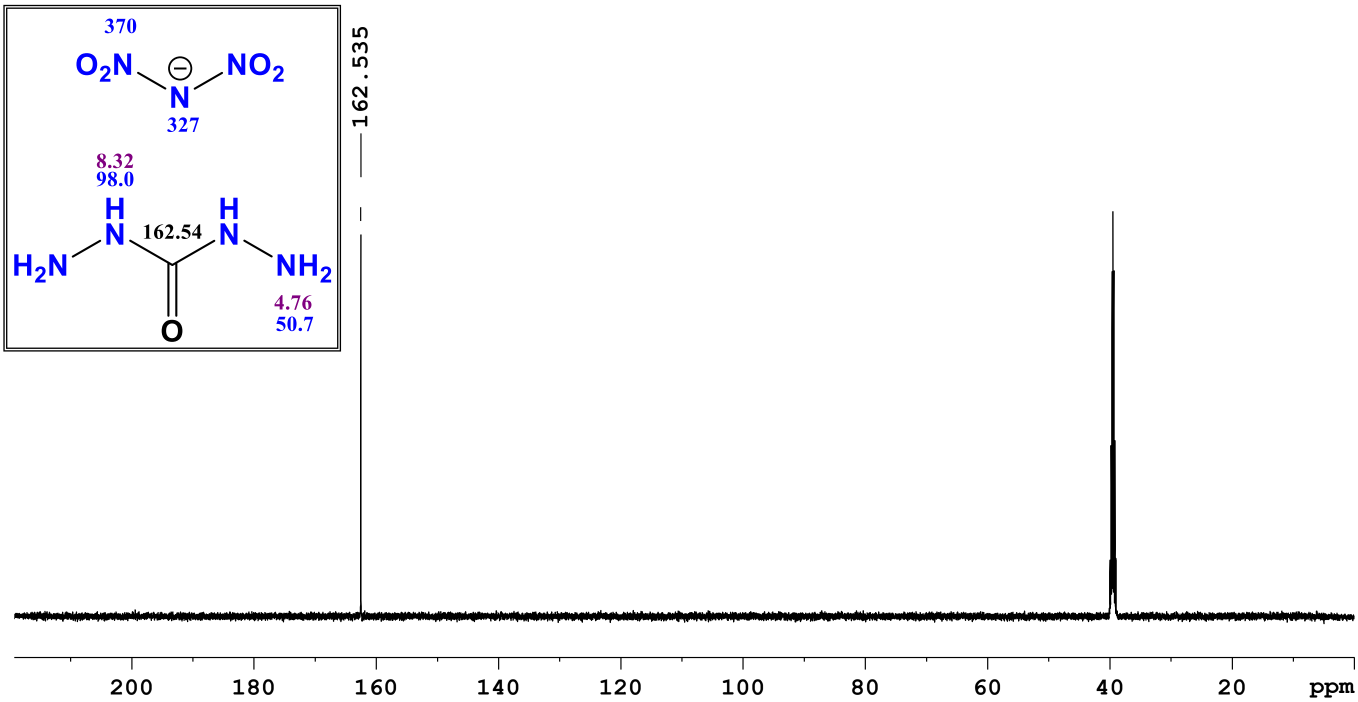

Figure S15. $^{13}C\{^1H\}$-NMR spectrum of **6**

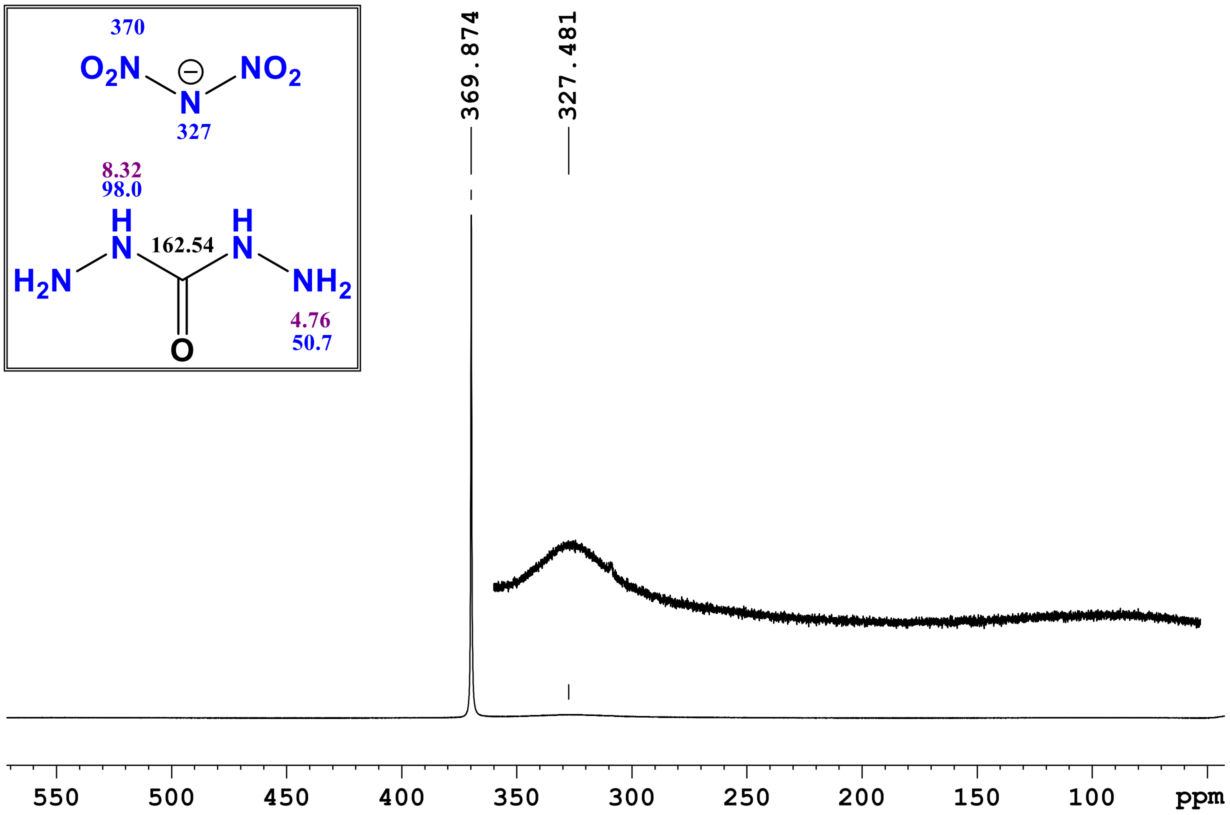

Figure S16. ^{14}N-NMR spectrum of **6**

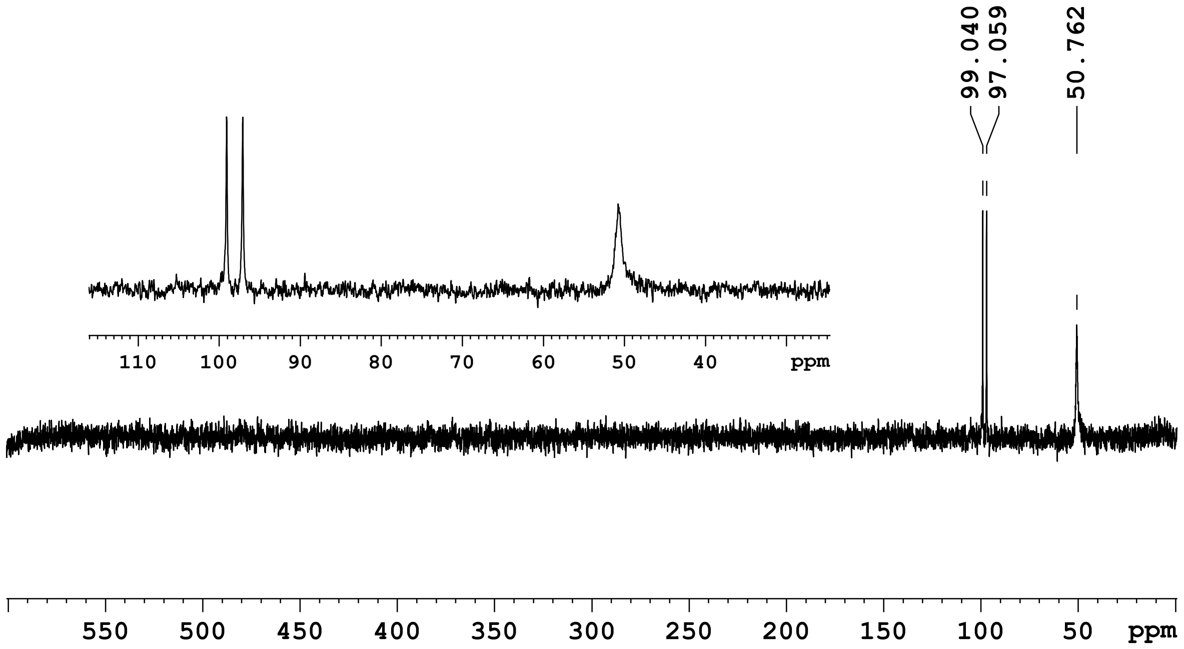

Figure S17. ^{15}N-NMR spectrum of **6**

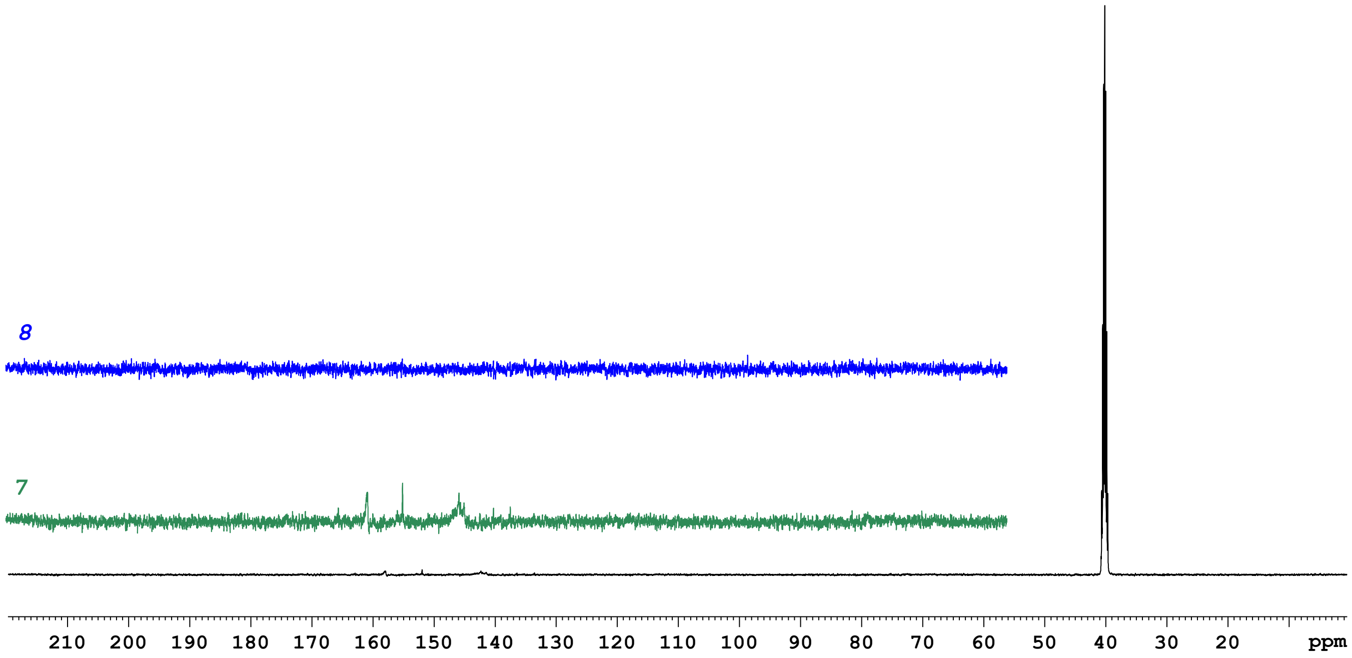

Figure S18. $^{13}C\{^{1}H\}$-NMR spectra of **7** and **8**. The green and blue frames are enlarged 8 times

The ^{1}H NMR spectra of both samples are alike. A good deal of signals belonging to different functional groups are observed in the spectra. There may exist both aromatic atoms of hydrogen and those belonging to isolated double bonds. The signals present in the region above 8 ppm indicate the presence of mobile protons, including those coming from amino groups. It should be noted that because of the low solubility of the sample, the solution could have been enriched with impurity components. Therefore, one cannot speak about the identity between the solution composition and the solid phase.

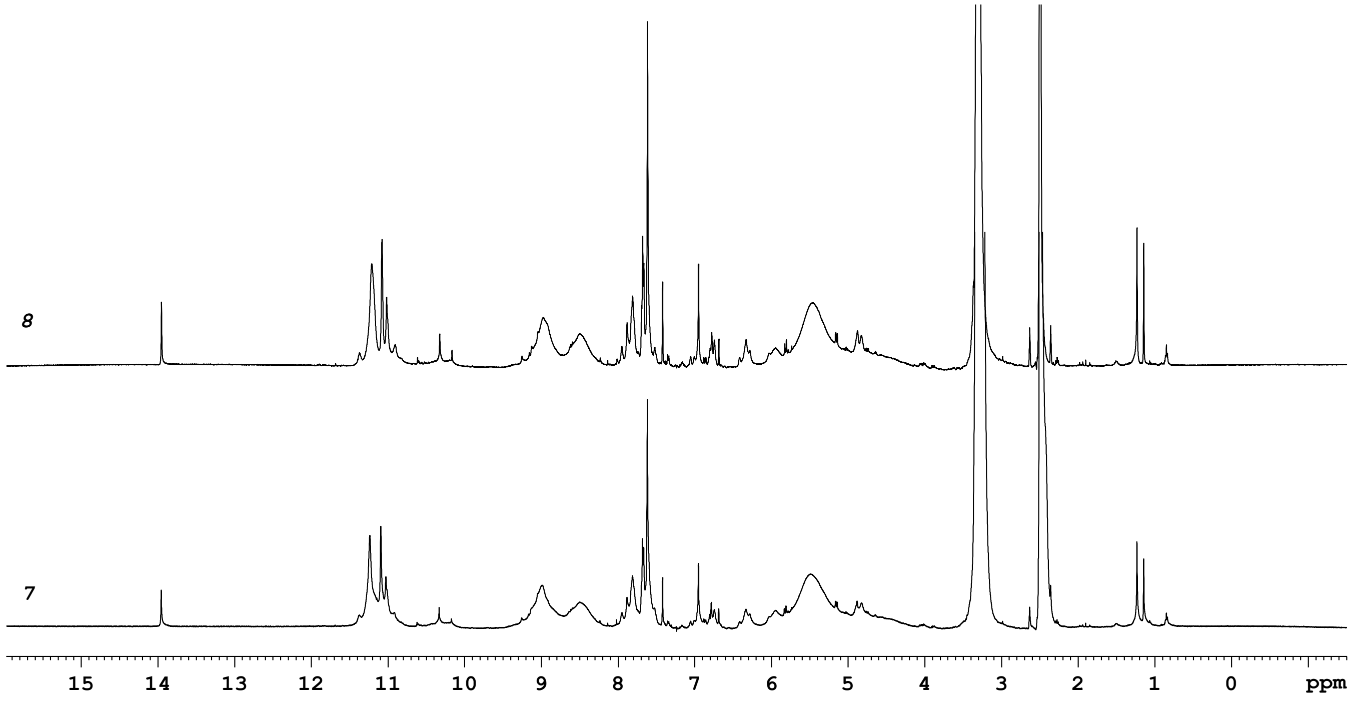

Figure S19. Comparison of the ^{1}H-NMR spectra of **7** (bottom) and **8** (top)

2D ¹H,¹H COSY NMR spectroscopy revealed several interactions between the signals. The cross-peaks between weak signals at 5.15 ppm (doublet of doublet, J_{HH} = 10.7 Hz, J_{HH}= 2.2 Hz) and 5.82 ppm (doublet, $^3J_{HH}$ = 10.7 Hz) called attention to themselves. They are well resolved but one should note that these multiplicities contradict the proposed structure and relate most likely to some impurity and/or byproduct. The 2D $^1H,^{13}C$ NMR correlation spectroscopy results allowed us to distinguish a series of CH pairs whose signals are concentrated near 140–142 ppm. An interaction between the NH hydrogen atoms and the carbonyl carbons was detected at 150 ppm. All of this allows us to assume a possible existence of the expected compound **2** in the solution. However, it proves impossible to check if carbohydrazide or dinitramide exists in the solution by the NMR technique, given the available solubility of the samples.

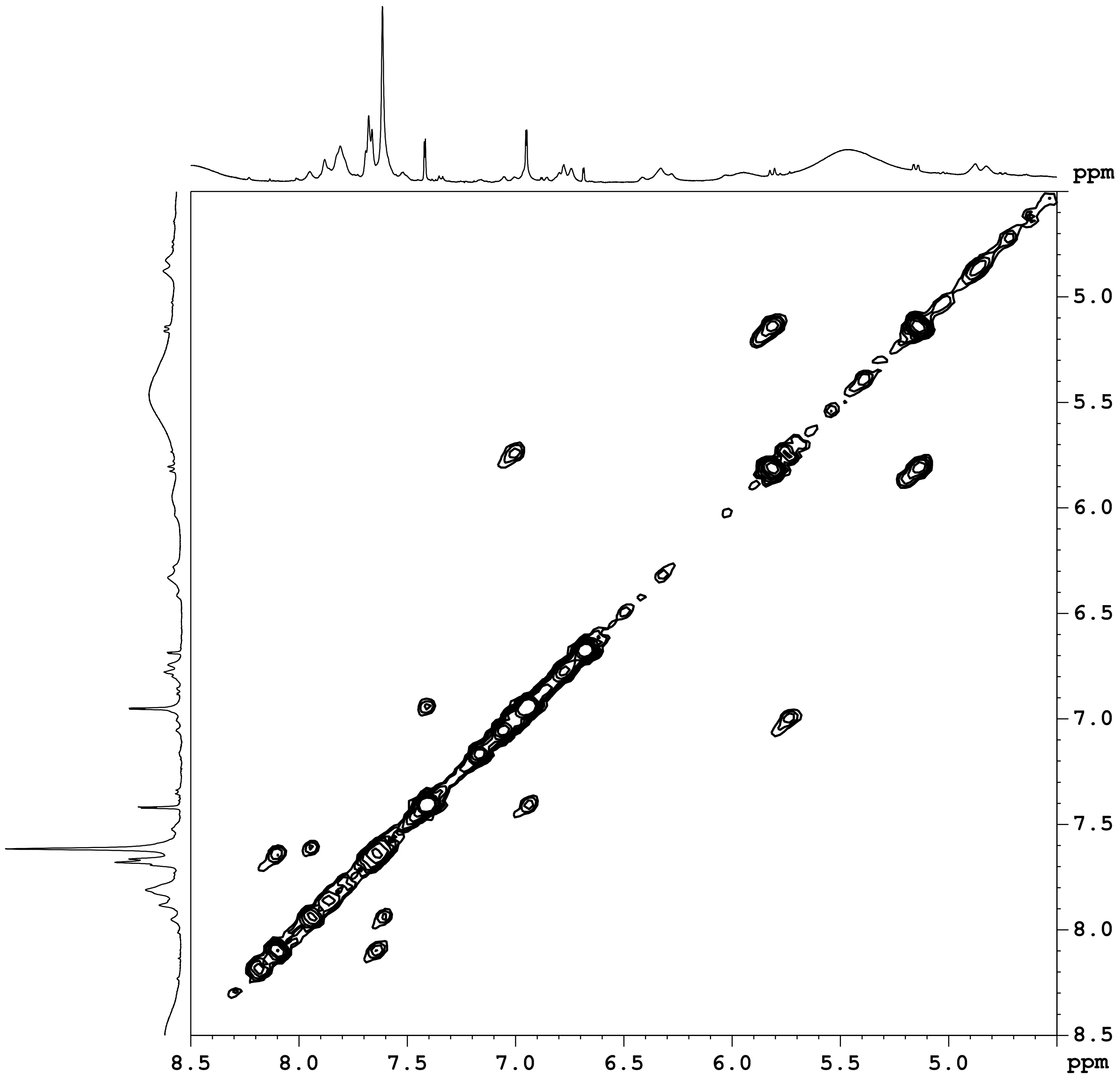

Figure S20. $^1H^1H$-COSY spectrum of **8**

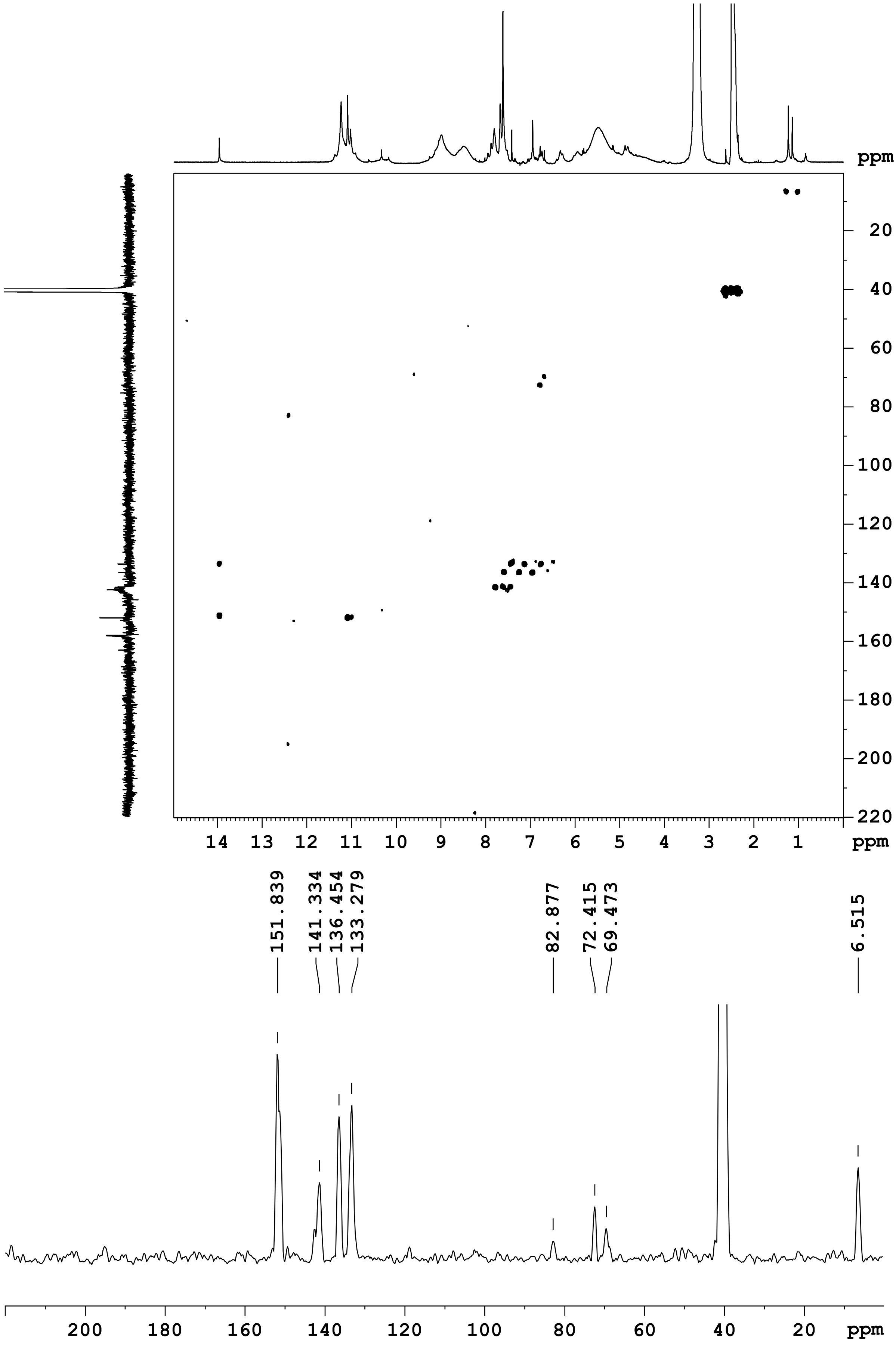

Figure S21. $^{1}H^{13}C$-HMBC spectrum of **7** and ^{13}C axis projection
